# Poromechanical toughening in tensile fracture of saturated solids

**DOI:** 10.1038/s41467-026-76352-3

**Published:** 2026-08-18

**Authors:** Antoine Guggisberg, Mehana Allache, Philipp Braun, Marie Violay, Mathias Lebihain

**Affiliations:** 1 Laboratory of Experimental Rock Mechanics (LEMR), ENAC, École Polytechnique Fédérale de Lausanne (EPFL), Lausanne, Switzerland; 2 Navier, ENPC, Institut Polytechnique de Paris, Université Gustave Eiffel, CNRS, Champs-sur-Marne, France

**Keywords:** Materials science, Geophysics

## Abstract

Fracture in fluid-saturated porous solids is governed not only by solid mechanics but also by fluid flow within the pore space. In systems ranging from geomaterials to biological tissues, deformation in the near-tip region perturbs pore pressure and thereby alters crack growth. Yet these pore-fluid effects have largely been inferred rather than directly observed. Here, we perform in situ pore-pressure measurements during controlled tensile rupture of a water-saturated microporous cement. We demonstrate that a propagating crack generates a localized underpressure ahead of its tip, providing a direct experimental signature of poromechanical coupling. The amplitude of this transient pressure drop scales with the square root of crack velocity, in agreement with poroelastic theory. This underpressure reduces the effective stress near the crack tip, leading to a coupled mechanical response: the apparent fracture energy doubles across the tested velocity range while the fracture process zone shrinks by half. Our findings reveal poromechanical coupling as a rate-dependent control on fracture in saturated porous materials, governed by effective stress changes induced by competing fluid diffusion and crack advance.

## Abstract (web summary)

Direct pore-pressure measurements show that tensile cracks in water-saturated cement generate local underpressure, increasing fracture resistance and reducing the size of the damage zone as crack speed rises.

## Introduction

Fracture in fluid-saturated porous solids is not governed by solid mechanics alone. As a crack propagates, deformation of the solid skeleton perturbs the fluid saturating the pore space, redistributing pore pressure and coupling local deformation with fluid flow^1–3^. This interaction occurs in diverse systems, from rocks under hydraulic stimulation^4–6^ to gels under tension^7^ and rupturing biological tissues^8^, and can exert a first-order control on rupture behavior^9–13^. However, the role of pore fluids during fracture remains largely inferred rather than observed: in most cases, fluid effects are absorbed into an apparent fracture resistance^7,14^, largely because pore-pressure transients are difficult to resolve at the spatial and temporal scales of crack propagation in the laboratory.

**Fig. 1:**
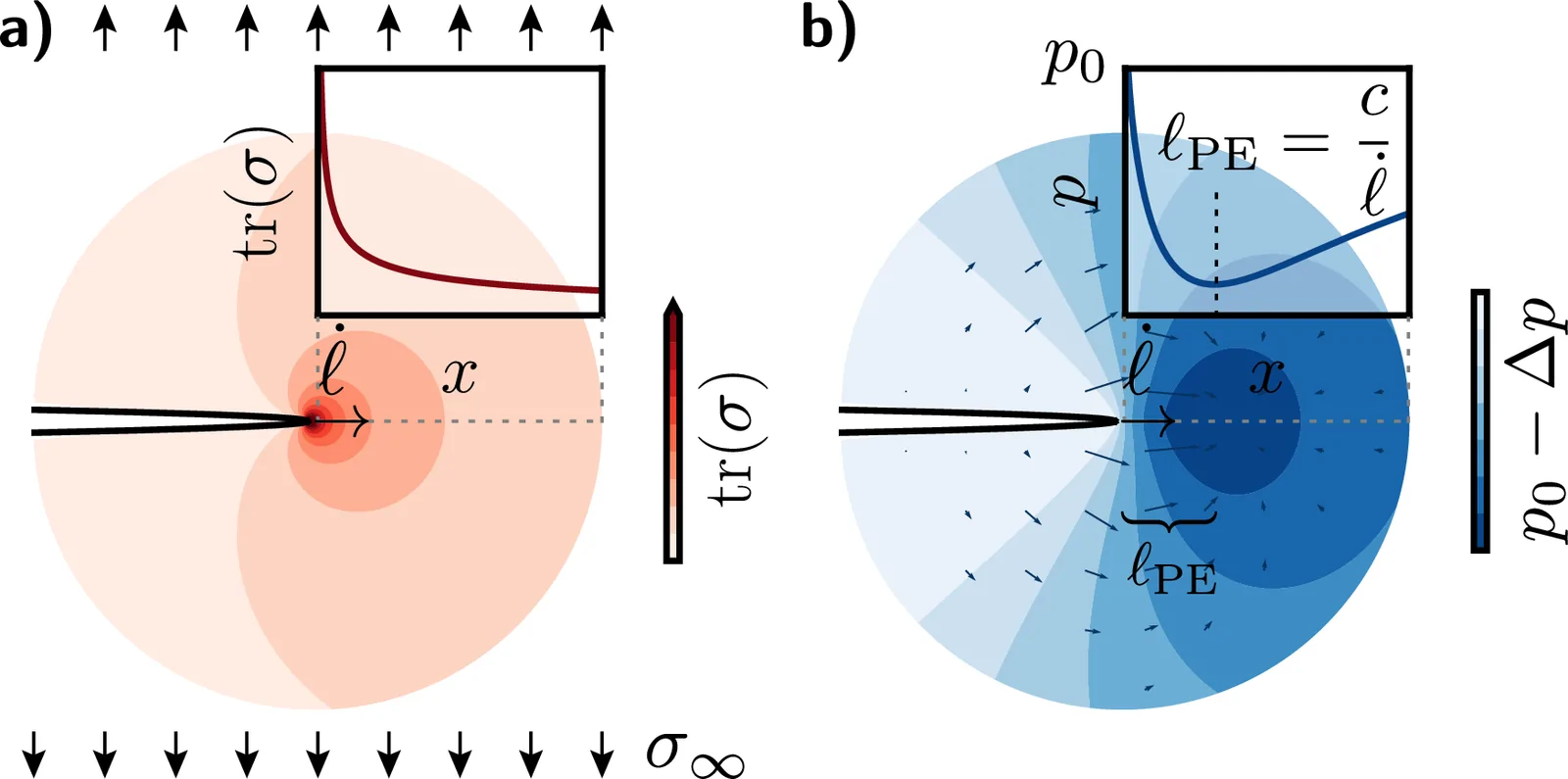
Poromechanical coupling during tensile crack propagation. **a** External loading concentrates stress *σ* at the crack tip, causing local dilation. **b** In saturated materials, this deformation draws fluid inward, generating a transient underpressure *Δp* ahead of the advancing tip, from an initial value *p*_0_. The spatial extent of this coupling is controlled by the poroelastic length scale ${\ell }_{{{\rm{PE}}}}=c/\dot{\ell }$ which depends on the material’s diffusivity *c* and the crack velocity $\dot{\ell }$.

In dry quasi-brittle materials, tensile crack propagation produces an intense stress concentration *σ* near the crack tip (Fig. 1a), activating irreversible mechanisms, such as yielding or damage, in the process zone *ξ*^15^. Resistance to crack growth is quantified by the fracture energy *G*_c_^16^, which corresponds to the energy dissipated within the process zone. In saturated porous materials, the concentrated dilation at the crack tip increases porosity, which draws fluid inward from the surrounding pores (Fig. 1b)^17–20^. This results in a transient drop of pore pressure *p* ahead of the crack tip, which reduces the effective stress ${\sigma }^{{\prime} }\simeq \sigma+p$ applied to the skeleton, modifies the near-tip failure micromechanisms, and ultimately leads to a change of apparent fracture energy *G*_c_ at the macroscopic scale. Ruina’s analysis^21^ showed that the magnitude and spatial extent of the pressure fluctuations are set by the competition between crack advance and fluid diffusion, captured by a single poroelastic length scale

1 $${\ell }_{{{\rm{PE}}}}=c/\dot{\ell },$$

where $\dot{\ell }$ is the crack velocity, and *c* is the hydraulic diffusivity, which controls the relaxation rate of the pressure variations in the porous solid. Rapid propagation yields an undrained regime, in which pore pressure cannot equilibrate quickly enough, leading to sharp, localized underpressures and strong poromechanical coupling, especially when *ℓ*PE falls below the process zone size *ξ*. In contrast, slow crack growth allows fluid drainage, resulting in diffuse pressure variations and weak coupling in a drained propagation regime. Indirect evidence for these poromechanical effects has been reported in soft materials such as hydrogels, where undrained crack growth manifests as a rate dependence of the fracture energy *G*_c_ in experiments^7,22–24^, supported by numerical simulations^25–28^, where chemical diffusion plays a role analogous to pore-pressure diffusion. However, in gels and biological tissues, the combination of low stiffness, low permeability and high fluid viscosity confines pore-pressure dynamics to micrometer and millisecond scales. Consequently, direct in situ observations of hydromechanical coupling, together with its mechanical consequences, remain limited.

To observe hydromechanical coupling involved in tensile rupture in the laboratory, we selected a rock analog material with a homogeneous pore structure, low viscosity and well-controlled mechanical and hydraulic properties: class G oil well cement^29–31^. In water-saturated conditions, its low permeability and high stiffness yield a hydraulic diffusivity of *c* ≃ 7.7 ⋅ 10^−7^ ⋅ m^2^/s. For laboratory-accessible quasi-static crack velocities, *ℓ*_PE_ spans micrometers to millimeters, thereby (i) enabling direct spatiotemporal resolution of fluid-solid interactions. Moreover, the material is quasi-brittle with a process zone size of *ξ* ≃ 0.6mm that overlaps accessible *ℓ*_PE_, which (ii) allows us to quantify fluid-induced variations in macroscopic fracture resistance and their rate dependence, in a regime where other rate effects (e.g., inertia or bulk viscous dissipation) are negligible (see Supplementary Material C). Finally, its fracture properties are shown below to be fairly insensitive to water exposure, thus (iii) excluding significant water-weakening and chemical effects that can be prevalent in geological and soft materials^32–36^, thereby isolating the role of poromechanical coupling.

To measure pore-pressure transients during crack growth, we developed a custom experimental platform that enables quasi-static stable crack propagation at prescribed velocity using a wedge splitting test (WST) in a water-filled triaxial pressurized cell^37^. A miniature pressure sensor embedded in the specimen provides real-time measurements of internal pore pressure. Combining crack opening displacement, measured via digital image correlation, with applied force, measured with a force sensor, we infer the crack velocity and fracture energy throughout the test.

Here, we show that a transient pressure drop develops ahead of the crack front, whose magnitude scales with the square root of crack speed, in agreement with poroelastic theory^20,21^. This underpressure reduces the near-tip effective stress, producing a fluid-induced toughening effect: the apparent fracture energy increases by up to a factor  ≃ 2 at the highest tested velocities. This toughening can be attributed to the contraction of the process zone, which leaves a measurable imprint on fracture surfaces, unraveled through green-light interferometry. Together, these findings identify *ℓ*_PE_ as a control parameter for fracture in saturated porous materials. By capturing the interplay between crack advance and fluid flow, *ℓ*_PE_ governs the emergence of toughening through localized dissipation and structural stiffening. More broadly, our results show that fluid-solid interactions can exert control over fracture processes with implications emphasized on geological formations but extending to hydrogels and biological tissues.

## Results and discussion

### A pressure drop generated by rapid crack propagation

Fracture tests were performed on a 135 × 80 × 25 mm class-G cement specimen, embedding a miniature pore pressure sensor connected to the rupture plane through a 0.5 mm-diameter conduit. The specimen was mounted in a wedge-splitting apparatus inside a water-filled triaxial cell (Fig. 5 in Methods). The setup enables continuous recording of pore pressure at 1kHz at a fixed location along the crack path, as the crack tip propagates past it at approximately constant velocity. Under locally steady propagation, the time series can be mapped onto the spatial evolution of pore pressure near the advancing tip. We conducted 27 experiments in total: seven with combined pore-pressure, crack velocities and computed fracture energy, 17 with pore-pressure only, and three dry reference tests with crack velocities and computed fracture energy (Supplementary Material A). The experiments covered $\dot{\ell }=0.2$-20mm/s over two orders of magnitude, corresponding to poroelastic lengths *ℓ*_PE_ spanning the transition from drained to undrained conditions (Supplementary Material C).

**Fig. 2:**
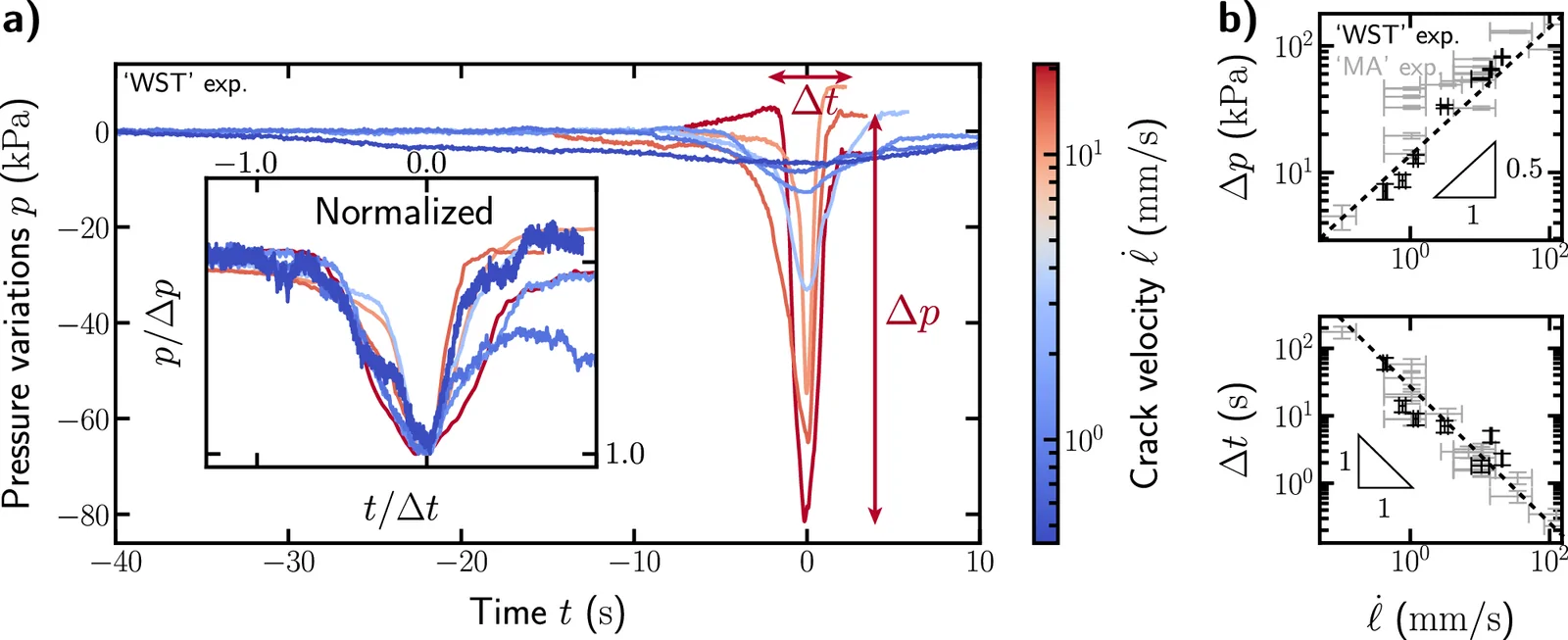
Pressure drops induced by crack propagation. **a** Pore pressure measured by the embedded sensor during seven wedge splitting tests (`WST') at various crack velocities showing a transient underpressure as the crack passes. When normalized by their respective amplitudes and durations, the signals collapse onto a self-similar curve indicating a generic response. **b** Scaling of pressure drop amplitude *Δp* and duration *Δt* with crack velocity $\dot{\ell }$. Error bars show propagated measurement uncertainty. Amplitudes increase as $\Delta p\propto {\dot{\ell }}^{0.5}$, while durations scale as $\Delta t\propto {\dot{\ell }}^{-1}$ (see Supplementary Material A for `MA' experiments, additional tests with only pore pressure measurements).

In all experiments, a transient underpressure developed as the crack front approached and passed the sensor (Fig. 2a and Supplementary Material A). Its amplitude Δ*p* ranged from 4.5 to 147.4 kPa and its duration Δ*t* from 0.2 to 173.8 s, depending on crack velocity. Δ*p* increased with crack velocity $\dot{\ell }$, while Δ*t* decreased. When normalized by their respective amplitudes and durations, the signals collapse onto a single self-similar curve (Fig. 2a inset), indicating a generic response parametrized by crack velocity. Note that the pressure drop observed in our experiments relates to the porous nature of the cement. This mechanism is distinct from the fluid lag of tensile hydraulic fractures propagating in impermeable media, where a zero-pressure gap can develop between the fluid front and the crack tip^38–40^.

We find that $\Delta p\propto \sqrt{\dot{\ell }}$ and $\Delta t\propto 1/\dot{\ell }$ (Fig. 2b). The scaling of Δ*p* agrees with theoretical poroelastic predictions for a steadily propagating tensile crack^20,21^, for which $\Delta p \sim {K}_{{{\rm{I}}}}\sqrt{\dot{\ell }/c}$ (Supplementary Material Eq. 13), where *K*_I_ is the stress intensity factor. This scaling links the pressure drop to the size *ℓ*_PE_ of the drained zone near the tip. However, absolute amplitudes are two orders of magnitude smaller than predicted, due to attenuation by the compliant dead volume of the pressure sensor, a common artifact during in situ poromechanical measurements^41^, and limiting assumptions of the theoretical model (see Supplementary Material C for detailed discussion C.4). The duration scaling is less steep than the analytical prediction $\Delta t \sim {\ell }_{{{\rm{PE}}}}/\dot{\ell } \sim c/{\dot{\ell }}^{2}$, likely due to experimental limitations (fluid damping within the dead volume, finite sample thickness and finite initial crack length) and physical effects that are not captured by the model (e.g., permeability enhancement in the process zone and fluid exchange through crack lips), all of which broaden and smooth the transient response. Nonetheless, the data confirm that rapid crack propagation in saturated porous solids produces a localized, velocity-dependent underpressure. Similar pressure variations have been shown to affect tensile crack propagation through a change in effective stress^11^.

### Fluid underpressure induces toughening

We now examine how the pressure drop influences the apparent fracture energy *G*_c_, obtained from the measured force *F* and crack length *ℓ* using the finite-element-based calibration curves (see Supplementary Material B). Here, *G*_c_ is not the intrinsic fracture energy of the cement skeleton, but the fracture energy that would be inferred if the saturated specimen were treated as an ordinary elastic solid^28^. It therefore lumps the hydromechanical response into a single apparent resistance, accounting for the dissipation generated in creating new surfaces, but also stress shielding by the pore pressure drop and dissipation in the fluid^42,43^.

**Fig. 3:**
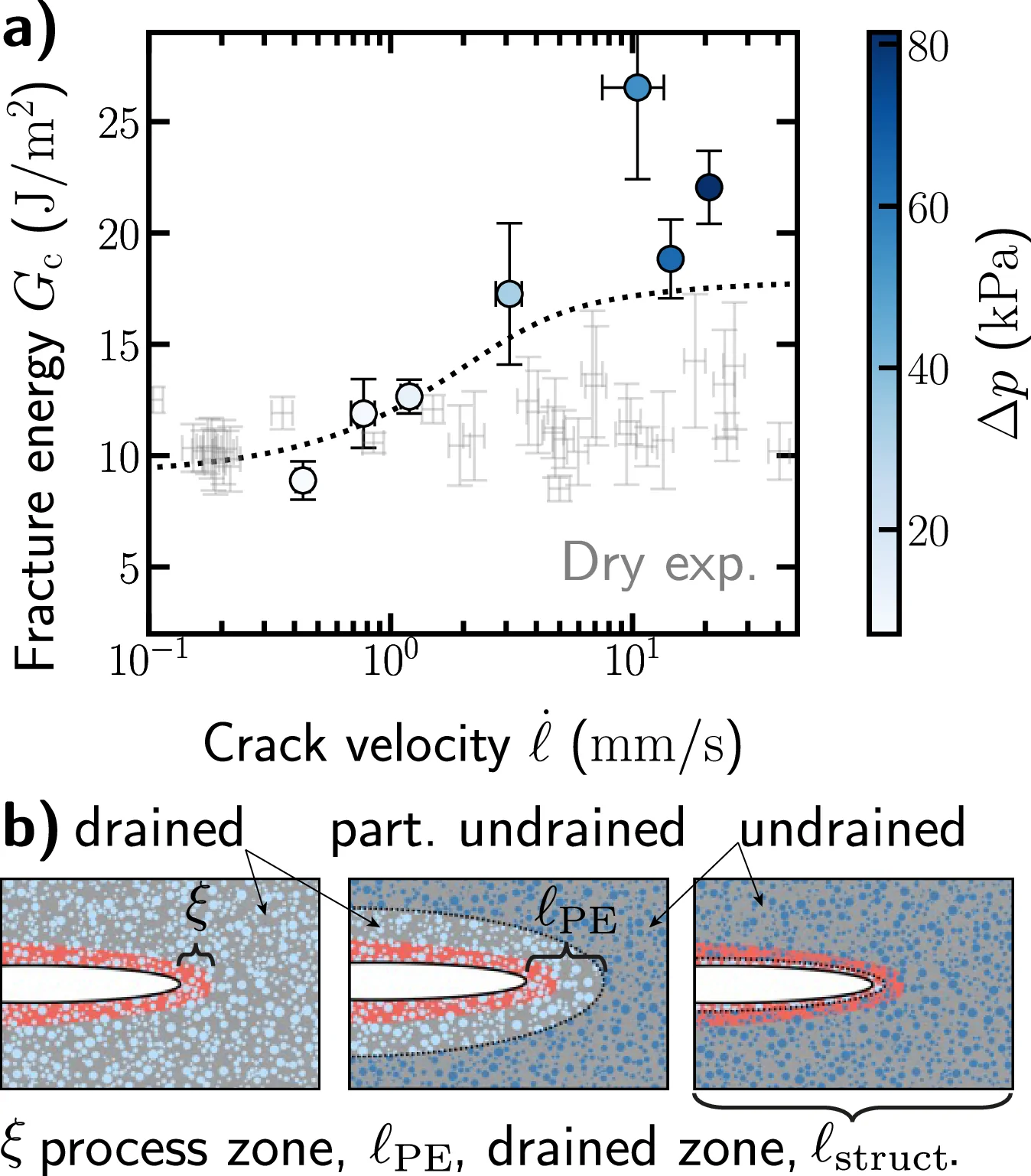
Velocity-dependent toughening from poromechanical coupling. **a** Fracture energy *G*_c_ as a function of crack velocity $\dot{\ell }$. Error bars show propagated measurement uncertainty. The dashed curve corresponds to Ruina’s analytical prediction for partially drained to undrained behavior^21^, evaluated using independently measured material parameters and the process zone size *ξ* = 0.6mm inferred from fracture surface analysis (see Supplementary Material C and D). Dry experiments (see Supplementary Material A.1) exhibit no velocity dependence. **b** The sigmoid shape reflects the progression from drained (*ℓ*_PE_ ≫ *ℓ*_struct_), to partially undrained (*ℓ*_struct_ ≫ *ℓ*_PE_ ≫ *ξ*), and finally to undrained regimes (*ξ* ≫ *ℓ*_PE_).

Our experiments show that *G*_c_ increases with crack velocity, doubling across the tested range (Fig. 3a, from 9 to 22 J/m^2^). These values were measured near the pressure sensor in the plateauing regime of crack propagation, when the process zone is fully formed and where both *G*_c_ and $\dot{\ell }$ are approximately constant. This increase cannot be attributed to dynamic or subcritical effects: all cracks propagate in a quasi-static regime, and importantly, dry reference experiments show no velocity dependence. Additionally, at low velocities, *G*_c_ values agree with literature for similar cementitious materials^44^, confirming the baseline behavior (also consistent with the absence of water weakening).

This velocity-dependent toughening arises from a gradual transition from drained to undrained conditions near the crack tip (Fig. 3b). According to Ruina’s poromechanical model^21^, two mechanisms contribute to the apparent increase in fracture energy. First, the drained zone of size *ℓ*_PE_ around the tip is more compliant than the surrounding undrained material, thereby reducing the local stress intensity factor and leading to an apparent toughening of up to 30% in cement. Second, under nearly undrained conditions, effective stresses ${\sigma }^{{\prime} }\simeq \sigma+p$ in the process zone are reduced, delaying failure and increasing the apparent fracture energy. Assuming an inelastic behavior where material degradation is controlled by the effective stress ${\sigma }^{{\prime} }$ rather than the total stress *σ*, this second mechanism can double *G*_c_.

The magnitude of these effects depends on the interaction between the poroelastic length scale ${\ell }_{{{\rm{PE}}}}=c/\dot{\ell }$, structural length scales *ℓ*_struct_ related to the specimen geometry (e.g., specimen dimensions, crack size, distance of application of forces), and the size of the process zone *ξ*. In our experiments, the diffusivity *c* ≃ 7.7 ⋅ 10^−7^m^2^/s and crack velocities span 0.2 to 20 mm/s, yielding values of *ℓ*_PE_ from 0.04 to 4mm. This range bridges both the specimen half-thickness (*ℓ*_struct_ ≃ 10mm), which governs global drainage, and the estimated process zone size (*ξ* ≃ 0.6mm, see next section). At low velocities, *ℓ*_PE_ ≃ *ℓ*_struct_, the material remains fully drained, and *G*_c_ matches the dry case. At high velocities, when *ℓ*_PE_ ≪ *ξ*, both poroelastic stiffening and effective stress reduction are in action, leading to the observed twofold toughening. The dashed curve in Fig. 3a corresponds to Ruina’s analytical prediction for the transition from partially drained to undrained regimes. It is evaluated using independently measured material parameters and the process-zone size *ξ* = 0.6 mm inferred from fracture surface analysis (see Supplementary Material C and D). Note that these predictions use the theoretical pore-pressure drop. Using the attenuated measured signals (biased by sensor dead volume) would largely suppress the apparent poroelastic toughening. Our experiments show that poromechanical coupling can significantly enhance the apparent fracture resistance in saturated brittle materials. Notably, the most pronounced toughening arises when the drained region of size *ℓ*_PE_ near the crack tip entirely contains the process zone, potentially reshaping the failure mechanisms.

### Damage localization induced by effective stress changes

Fracture surfaces preserve a persistent record of the failure processes active during crack propagation^45^. If poromechanical coupling redistributes effective stresses and alters the localization of damage, the fracture surfaces should retain a measurable imprint of these changes. To test this, we analyzed the roughness of post-mortem crack surfaces scanned near the pressure sensor using green-light profilometry. Across all conditions, the height fluctuations on the fracture surfaces exhibit two distinct roughness regimes (Methods; Supplementary Material D), identified from the scale-dependent statistics of height increments. At large (millimetric) scales, the surfaces display a self-affine regime typical of brittle fracture in linearly elastic materials^46^. At smaller scales, however, the roughness becomes increasingly influenced by localized near-tip damage processes, including microcrack nucleation and coalescence^47^. This small-scale regime exhibits complex height fluctuations with fat-tailed statistics^48,49^. The scale at which these fluctuations become statistically significant defines a characteristic crossover length $\tilde{\xi }$, which we interpret, following previous studies^48–53^, as a proxy for the spatial extent of damage processes within the process zone.

**Fig. 4:**
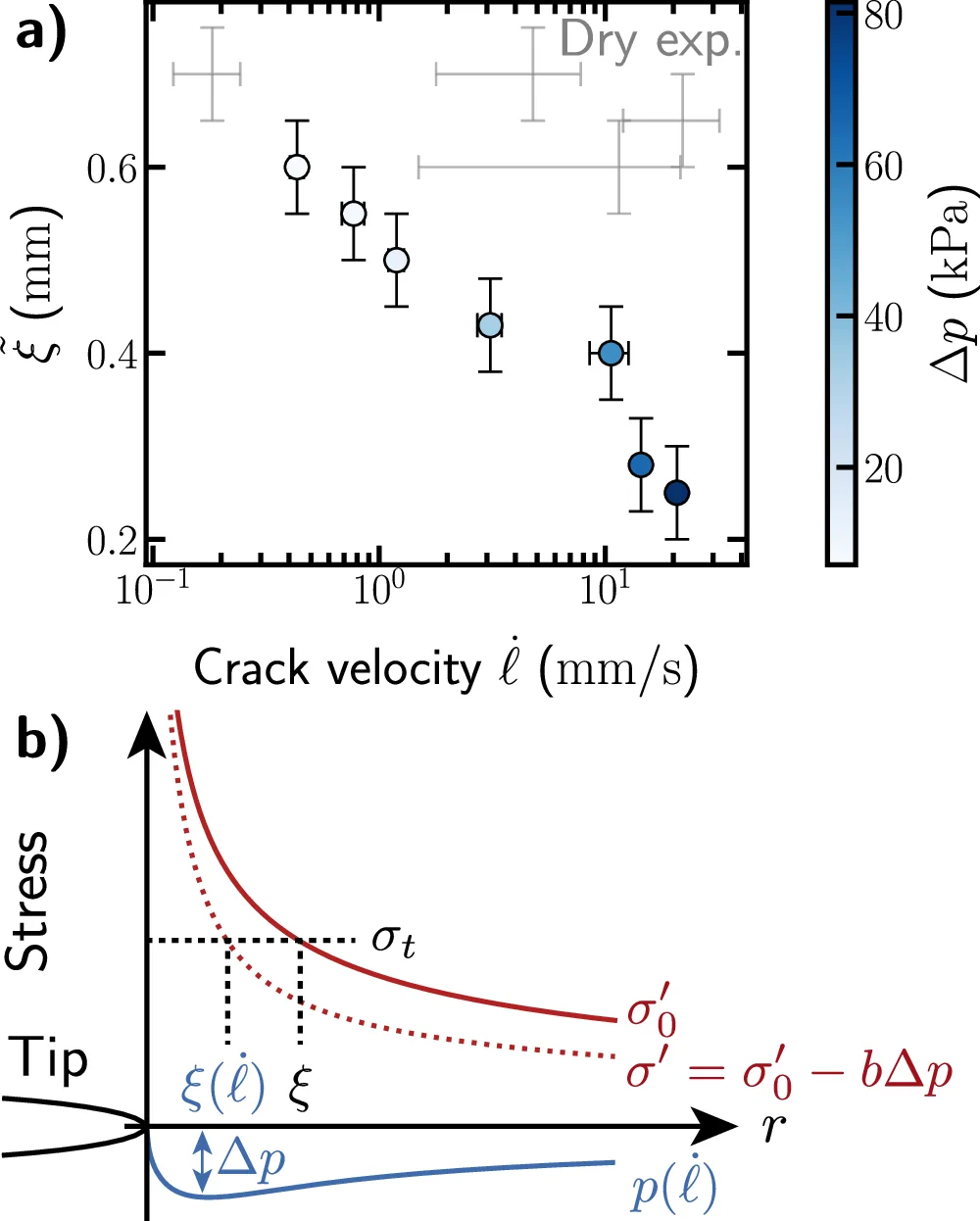
Apparent shrinkage of the process zone. **a** crossover length scale $\tilde{\xi }$ decreasing with increasing crack velocity $\dot{\ell }$ under saturated conditions only. Error bars show propagated measurement uncertainty. **b** a simple effective stress argument may rationalize. The pore pressure drop *Δp* from its initial value *p*_0_ reduces the effective stress ${\sigma }_{0}^{{\prime} }=\sigma+b{p}_{0}$ to ${\sigma }^{{\prime} }=\sigma+b{p}_{0}-b\Delta p$, potentially confining damage processes closer to the crack tip for a fixed yielding stress *σ*_*t*_.

In dry experiments, $\tilde{\xi }$ remains nearly constant at  ~ 0.6–0.7 mm (Fig. 4a), consistent with the expected order of magnitude of the Irwin length in cementitious materials, $E{G}_{{{\rm{c}}}}/(2\pi {\sigma }_{{{\rm{t}}}}^{2})\approx 1.1\,{{\rm{mm}}}$^54^, where *σ*_t_ denotes the tensile strength. In contrast, under saturated conditions, $\tilde{\xi }$ decreases systematically with increasing crack velocity from 0.6 to 0.25 mm (Fig. 4a).

This apparent shrinkage in saturated conditions can be understood from the fundamentals of porous media mechanics: pore pressure acts directly on the grains and thus modifies the stress carried by the solid skeleton^55,56^. Here, as the crack advances, the induced underpressure reduces the pore fluid pressure, thereby lowering the effective stress ${\sigma }^{{\prime} }=\sigma+bp$ acting on the matrix, where the Biot coefficient *b* ≃ 0.6 ^29^ quantifies the coupling between pore pressure and deformation of the solid skeleton. This delays the onset of local yielding, confining inelastic deformation failure mechanisms closer to the crack tip (Fig. 4b). The apparent reduction of the process zone in saturated conditions arises from the same effective-stress reduction mechanism that increases the apparent resistance to crack propagation. The same poromechanical coupling responsible for toughening may therefore also alter the spatial extent of near-tip damage.

### Poromechanics generalizes across porous solids

Our findings indicate that fluid flow ahead of a propagating rupture exhibits generic behavior that is well explained by poroelasticity and fracture mechanics. This coupling emerges from the interplay between fluid diffusion and crack advance, as evidenced by our direct measurements of a transient underpressure field ahead of a tensile crack. Importantly, our theoretical analysis indicates that this behavior is not specific to the tested cementitious material but rather a generic poromechanical response of saturated porous media. The phenomenon is governed by a single characteristic length scale ${\ell }_{{{\rm{PE}}}}=c/\dot{\ell }$, which compares fluid diffusivity to the crack propagation speed. As such, any saturated porous material, from rocks and concretes to hydrogels and biological tissues, is expected to exhibit similar behavior, but on different velocity ranges.

The extent of poromechanical coupling depends on how *ℓ*_PE_ compares with both the structural drained zone *ℓ*_struct_ and the process zone *ξ*. This interaction controls whether the material behaves in a drained, partially drained, or undrained regime. Because these length scales vary across materials and loading conditions, the onset of poromechanical effects is specific to each system. For instance, the comparatively large diffusivity of sandstones *c* ≃ 10^−4^ − 1m^2^/s^57^ makes poromechanical effects prevalent at much larger quasi-inertial crack speed $\dot{\ell }\simeq 1-1000\,{{\rm{m/s}}}$. In contrast, hydrogels *c* ≃ 10^−10^m^2^/s ^24^ experience undrained poromechanical effects even at subcritical crack growth rates, although other viscous dissipation mechanisms may dominate at higher velocities^58^. These rate-dependent pressure transients have direct mechanical consequences for the stress state governing material failure.

While fracture energy remained constant in dry conditions, it became velocity-dependent under saturation, doubling from 9 to 22 J/m^2^ with increasing quasi-static crack velocity. This behavior aligns with the predicted emergence of two toughening mechanisms^21^: (1) a drained response at the crack tip, which lowers the effective elastic moduli and increases the apparent fracture energy by up to 30%, and (2) an effective stress reduction in the process zone, which delays yielding and increases fracture energy by a factor of two (Supplementary Material C). Similar velocity-dependent toughening has been reported in saturated rocks, where conditions promoting an undrained response, such as higher fluid viscosity^59,60^ or faster crack propagation^61^, produce larger fracture energies. In geological settings, such toughening variations can be comparable in magnitude to heterogeneity contrasts between lithologies, and may significantly affect crack propagation dynamics and stability^62^. The poromechanical toughening depends on how strongly pore pressure couples to the solid skeleton, as quantified by the Biot coefficient *b*. In our cementitious material, the coupling is moderate, but in materials such as hydrogels, biological tissues, or low-permeability shales, it is stronger, potentially increasing apparent fracture energy by an order of magnitude or more (Supplementary Material C).

These poromechanical effects have direct implications for the stability of tensile rupture under dynamic and cyclic (un)loading. At high crack speeds, the increase in effective fracture energy acts as a brake, slowing down dynamic rupture. Under cyclic loading, the competition between loading time scales and hydraulic diffusion introduces a frequency dependence^63^. When the cycle duration *T*_cycle_ is much smaller than the diffusion time at the process zone scale *T*_diff_ = *ξ*^2^/*c*, the undrained response of the process zone produces a strong poromechanical shielding, limiting crack advance per cycle^64^. When *T*_cycle_ ≫ *T*_diff_, the shielding is weaker, promoting long-term fatigue. In very low-permeability materials, unloading may generate a transient increase in pore pressure^63,65^, similar to stress-induced pressurization^17,66^. As such, short, rapid pressure pulses could use poromechanical toughening to promote distributed microcracking and permeability enhancement while limiting intermittent ruptures, whereas slow or long-duration pressure changes in a more permeable region may instead favor larger crack extensions.

In our cementitious system, poromechanical toughening is primarily driven by effective stress reduction, as evidenced by both the twofold increase in apparent fracture energy at the macroscopic scale and the shrinkage of the process zone from $\tilde{\xi }=0.6$ to 0.25 mm. These effects are likely dominant when pore pressure is close to the confining stress, as in overpressurized reservoirs. Poromechanical coupling may activate additional mechanisms, e.g., poroelastic backstress when fluid flow from the fracture to the medium is prevalent^10,67^, fluid-driven advection of reactive species, which can accelerate damage in chemically active systems^7,27,28^. To avoid cavitation during our experiments, we applied a confining pressure of 500 kPa. Still, cavitation is thought to occur in systems ranging from hydraulic fracturing and granular failure to brain tissue rupture and hydrogel fracture^8,68–71^, and remains largely unexplored in fracture experiments, calling for further investigation.

By linking in situ pore-pressure transients, fracture energy, process zone size, and crack velocities, we show how fluid-solid interactions reshape classical fracture mechanics and govern the transition between drained and undrained regimes at the process zone scale. These insights provide a common basis for interpreting and modeling fracture in materials as diverse as cements, geomaterials, biological tissues, and hydrogels. They point towards rate- and drainage-aware design strategies for natural and engineered systems in which tensile rupture, cyclic loading, and fluid flow are strongly coupled.

## Methods

**Fig. 5:**
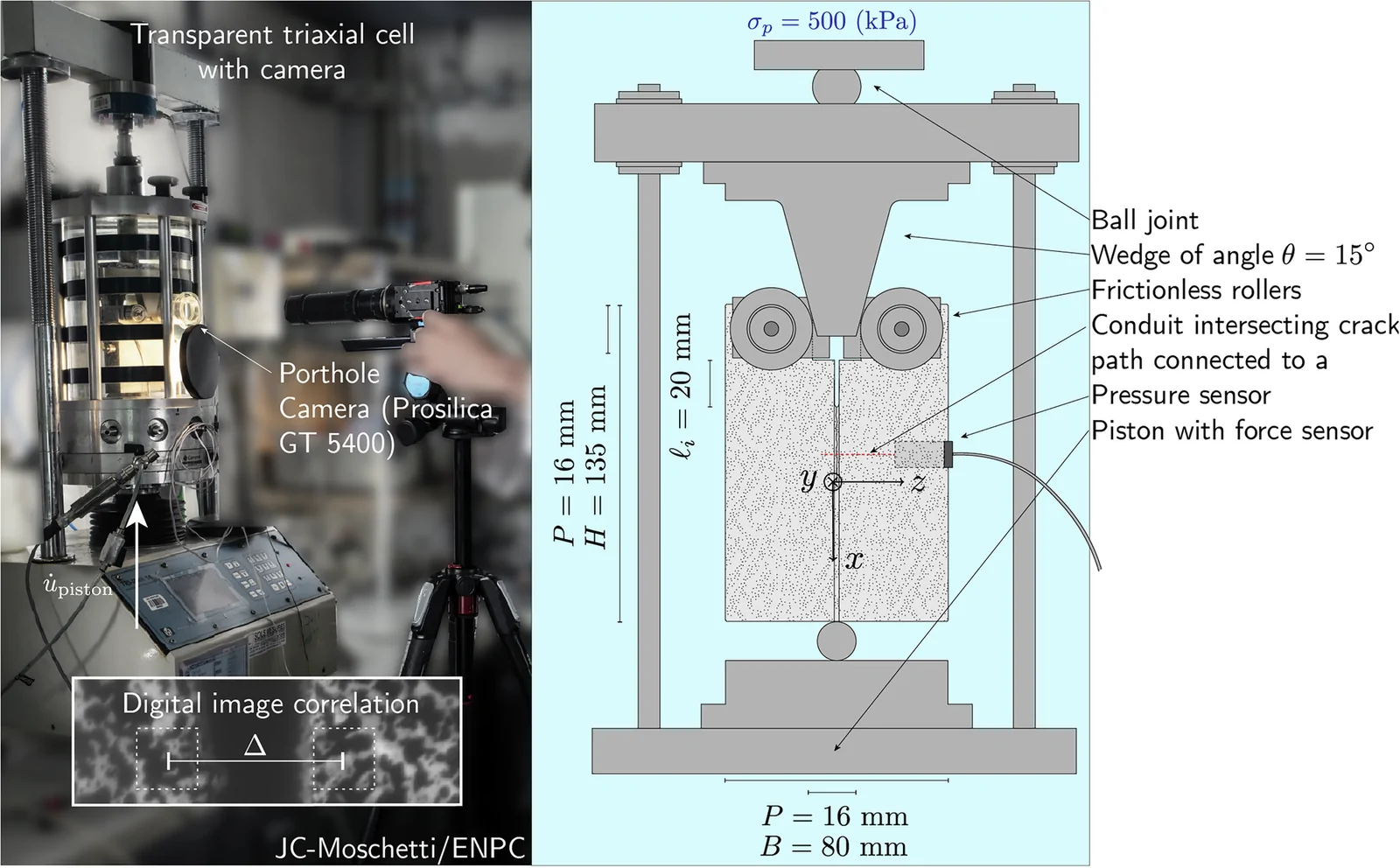
Testing geometry: wedge splitting test (WST) adapted for use in a triaxial cell at *σ*_*p*_ = 500 kPa. The vertical press drives a wedge applying 15^∘^ splitting forces via frictionless rollers. Crack mouth opening is measured by digital image correlation (DIC) through a porthole. A sealed pore pressure sensor embedded in the specimen records pressure variations in a 0.5 mm-diameter conduit intersecting the crack path.

A wedge splitting test (WST) was adapted for use in a water-filled pressurized cell, enabling simultaneous measurement of fracture energy, crack velocity, and internal pore pressure during quasi-static tensile fracture of saturated porous specimens (Fig. 5).

Specimens were 135 mm-long, 80 mm-wide, and 25 mm-thick blocks of cementitious material. They were cast from class G oil well cement (water-cement ratio 0.45, 0.37% dispersant) in 3D-printed molds, with a 30° V-notch (20 mm deep) and two 4 mm-deep guiding grooves to promote crack growth along a straight fracture path. They incorporated a cavity for a diaphragm-type miniature pore-pressure sensor (KYOWA PS-50KDM2, 2MPa capacity). The cavity is located 20 mm downstream of the notch and 21 mm away from the fracture plane to limit path deviations due to local stiffness contrasts. The sensor diaphragm was connected to the fracture plane by a 0.5 mm-diameter channel. Lateral sensor-cavity interfaces were sealed with epoxy resin. Samples were water-cured for one week. All loading faces, notches, and grooves were finish-machined for dimensional consistency. Crack propagation was predominantly straight through the thickness, with only minor out-of-plane deviations. The hydromechanical properties of the material have been measured in previous studies^29–31^: it has a low permeability *k* ≃ 10^−19^m^2^ and a high stiffness *E* ≃ 11.8 GPa, which yield a hydraulic diffusivity for water of *c* ≃ 7.7 ⋅ 10^−7^ ⋅ m^2^/s.

Specimens were mounted on the modified WST inside a water-filled triaxial pressurized cell. A constant isostatic pressure of 500 kPa was applied using a GDS Instruments high-pressure/volume controller and controlled using a pressure sensor (LucasSchaevitz pressure transducer, 1.5 MPa capacity). The pressure was maintained for several hours before testing to ensure saturation (Supplementary Material A.3). The wedge was driven by the vertical piston of a VJT TriSCAN 50kN frame, at constant displacement rates between 0.01 and 2 ${{\rm{mm/min}}}$, applying a 15° splitting force via frictionless rollers. A 16 mm shaft accommodated the loading supports. Normal force was measured using a GDS Instruments internal submersible load cell (5kN capacity). Axial force, confining pressure, and internal pore pressure were sampled at 1 kHz. Crack mouth opening was measured by digital image correlation (DIC) using a high-resolution camera (Prosilica GT 5400) through a porthole in the cell to minimize distortion. Camera acquisition was synchronized with mechanical data via a load-based trigger.

A total of 27 tests were performed: seven fully instrumented, 17 with pore-pressure measurements only, and three dry-reference tests with fracture energy and crack velocity measurements only. The loading conditions and test results of each test are reported in Supplementary Table 1.

Crack length *ℓ* and apparent fracture energy *G*_c_ were obtained using a compliance-based method^37^, in which experimental compliance is matched to finite-element (FE) calibration curves^72^. This analysis assumes both linear elasticity and small-scale yielding. In particular, no correction was applied to the measured pore-pressure transient. Instead, the effect of pore pressure on the load-crack response is deliberately retained in the apparent fracture energy *G*_c_ (see Supplementary Material B). FE simulations were performed using the Cast3M solver on half the WST geometry, owing to symmetry considerations. The geometry was meshed using Gmsh^73^ with second-order T6/Q8 elements. The simulations were run for a unit displacement *u* = 1 under plane-stress conditions, unit Young’s modulus, and Poisson’s ratio *ν* = 0.3, representative of the tested cement. Normalized compliance *λ* and normalized energy release rate *g* were computed, with *g* obtained from a *J*-integral^74^. The displacement orientation was adjusted to maintain a 15° force angle, assuming frictionless rollers. Experimental compliance *Λ* = Δ/*F* and energy release rate *G* were obtained from the nondimensional FE functions via *Λ* = *λE* and *G* = *gEu*^2^, where *E* is measured from the elastic part of the loading curve and *u* is the opening displacement retrieved from digital image correlation. Data after the onset of out-of-plane deflection or dynamic events were excluded. Measurement uncertainties were evaluated by error propagation of the individual measurement errors (Supplementary Material B).

Post-mortem fracture surfaces were scanned with a green-light profilometer (Bruker Contour GT-K) over 5 × 5 mm^2^ areas near the pressure conduit, at 1.97 μm pixel resolution. Height fluctuation statistics ${\langle | \delta z{| }^{q}\rangle }^{1/q}$ were computed versus spatial lag *δr* following established methods^48–50^ to obtain the scale-dependent Hurst exponent *H*_*q*_. The crossover from a *q*-dependent (multi-affine) to a *q*-independent (mono-affine) *H*_*q*_ defines the characteristic length *ξ* (Supplementary Material D).

## Supplementary information

Supplementary Material

Transparent Peer Review file

## Data Availability

The processed experimental data underlying the figures have been deposited in Zenodo under https://doi.org/10.5281/zenodo.18891332^75^.
