# Supplementary material for: Poromechanical toughening in tensile fracture of saturated solids
Source: Nat Commun. 2026 Aug 18;17:9890. doi: 10.1038/s41467-026-76352-3 (PMC13582804; doi:10.1038/s41467-026-76352-3)
Supplement: Supplementary file 1 [file 41467_2026_76352_MOESM1_ESM.pdf]

Supplementary Material for Poromechanical Toughening in Tensile Fracture of Saturated Solids

Antoine Guggisberg*[1], Mehana Allache[2], Philipp Braun[2], Marie Violay[1], and Mathias Lebihain[2]

[1]Laboratory of Experimental Rock Mechanics (LEMR), ENAC, École Polytechnique Fédérale de Lausanne (EPFL), Lausanne, Switzerland
[2]Navier, ENPC, Institut Polytechnique de Paris, Université Gustave Eiffel, CNRS, Champs-sur-Marne, France

This Supplementary Material provides:

A. the detailed experimental results (experimental array, dry experiments, supplementary experiments, validation of pore pressure equilibration),

B. their mechanical analysis (compliance method),

C. the poroelastic framework and scaling analysis (material parameters, characteristic length scales, analytical solution, and toughening regimes),

D. and the post-mortem fracture surface analysis (surfaces, fractal analysis).

*Corresponding author: antoine.guggisberg@epfl.ch

A Full experimental dataset

Supplementary Table 1 lists the main measurements from all 27 tests. "WST" refers to fully instrumented wedge-splitting tests, "Dry" to partially dried reference experiments, and "MA" to tests with pore-pressure measurements only.

Supplementary Table 1: Array of experiments with main measurements.

Experiment name	Piston velocity $\dot{v}$ (mm/min)	Crack velocity $\dot{\ell}$ (mm/s)	Fracture energy G_c (J/m^2)	Pressure drop Δp (kPa)	Drop duration Δt (s)
WST05	0.05	0.43	8.9	7.1	61.7
WST10	0.5	3.1	17.3	33.1	4.5
WST26	1	14.4	18.8	65.0	3.4
WST27	2	10.5	26.5	54.7	1.1
WST31	0.2	1.2	12.7	12.8	11.9
WST32	0.1	0.77	11.9	8.6	19.9
WST33	2	20.7	22.1	81.5	3.1
Dry04	0.5	0.8 - 13.4	10 - 12	-	-
Dry07	2	3.7 - 40.6	9 - 14	-	-
Dry08	0.05	0.08 - 0.36	10 - 13	-	-
MA-01	3.33	-	-	126.7	0.6
MA-02	3.33	-	-	131.2	1.2
MA-03	0.1	-	-	46.1	8.9
MA-04	1	-	-	59.7	1.5
MA-05	1	-	-	77.3	3.2
MA-06	0.66	-	-	52.7	2.9
MA-07	0.33	-	-	49.2	10.6
MA-09	0.1	-	-	32.6	57.7
MA-10	0.1	-	-	39.6	36.1
MA-13	0.01	-	-	4.5	173.8
MA-15	1	-	-	32.2	2.9
MA-16	0.1	-	-	19.5	18.9
MA-17	0.1	-	-	14.0	20.8
MA-19	12	-	-	93.6	0.3
MA-20	1	-	-	68.4	1.6
MA-21	1	-	-	53.6	2.4
MA-22	20	-	-	147.4	0.2

A.1 Dry experiments

3 partially dried ("pseudo-dry") tests were conducted to provide baseline fracture properties without pore fluid effects. Achieving fully dry conditions without premature cracking was not feasible due to desiccation-induced damage. Instead, specimens were partially dried to 50% saturation (one day after casting, one week of underwater curing, followed by two weeks at 0 to 30% relative humidity). Saturation was measured gravimetrically. This protocol prevents desiccation cracking while ensuring the pore network is no longer saturated. In these experiments, the pore pressure sensor was removed, and crack mouth opening was measured directly using an extensometer.

Supplementary Fig. 1a shows stable desiccation curves. WSTs were performed at piston velocities of 0.05, 0.5, and 2 mm/min (Supplementary Fig. 1b), from which fracture energy G_c and crack velocity $\dot{\ell}$ were computed over 5 mm propagation windows (Supplementary Fig. 1c). There is no dependency of fracture energy with crack velocity in dry conditions.

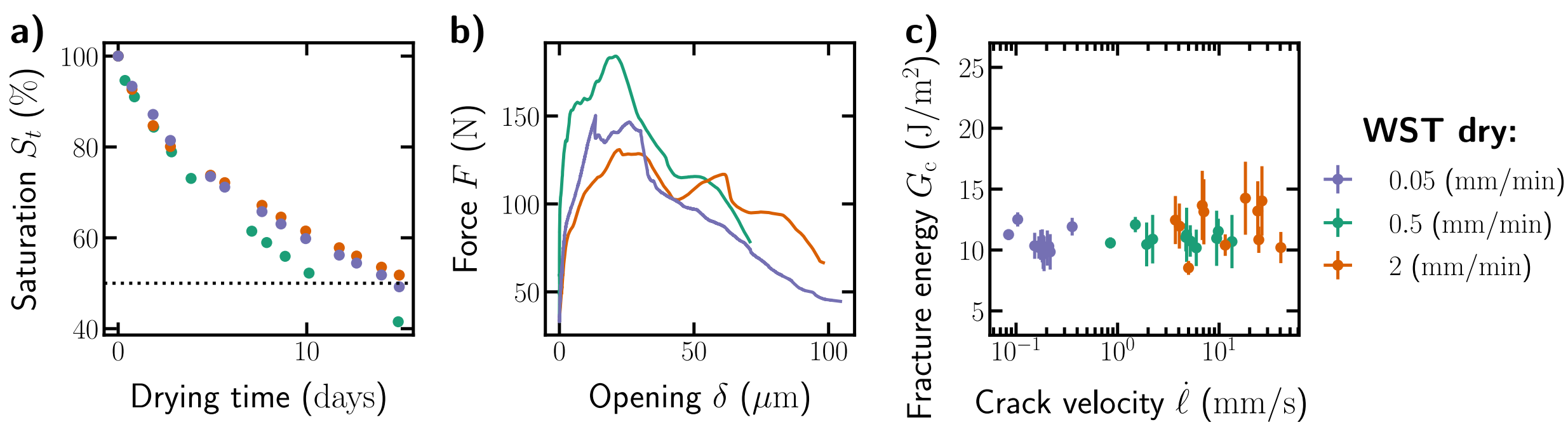

Supplementary Figure 1: Dry experiments: a) desiccation curves, b) mechanical measurements, and c) fracture energy G_c showing no dependence on crack velocity $\dot{\ell}$. Error bars show propagated measurement uncertainty.

A.2 Pore pressure-only tests

19 additional tests recorded only internal pore pressure signals to study the scalings of their features. The pressure profiles (Supplementary Fig. 2) display velocity-dependent trends consistent with those observed in the fully instrumented tests. As in all experiments, we define Δp as the amplitude of the transient drop relative to the average initial undisturbed pressure. To determine the drop duration Δt, we locate the intersection of the initial baselines with an affine best fit to the linear portion of the drop (between 10% and 60% of Δp). Δt is the time interval between the intersections of the onset and the recovery.

Since crack mouth opening was not measured, $\dot{\ell}$ could not be obtained directly. Instead, crack velocity was estimated from the piston velocity using the average ratio $\dot{\ell}/\dot{v} \approx 500$ measured in the fully instrumented tests, consistent with the LEFM compliance analysis (250–1000 range; Supplementary Fig. 5b).

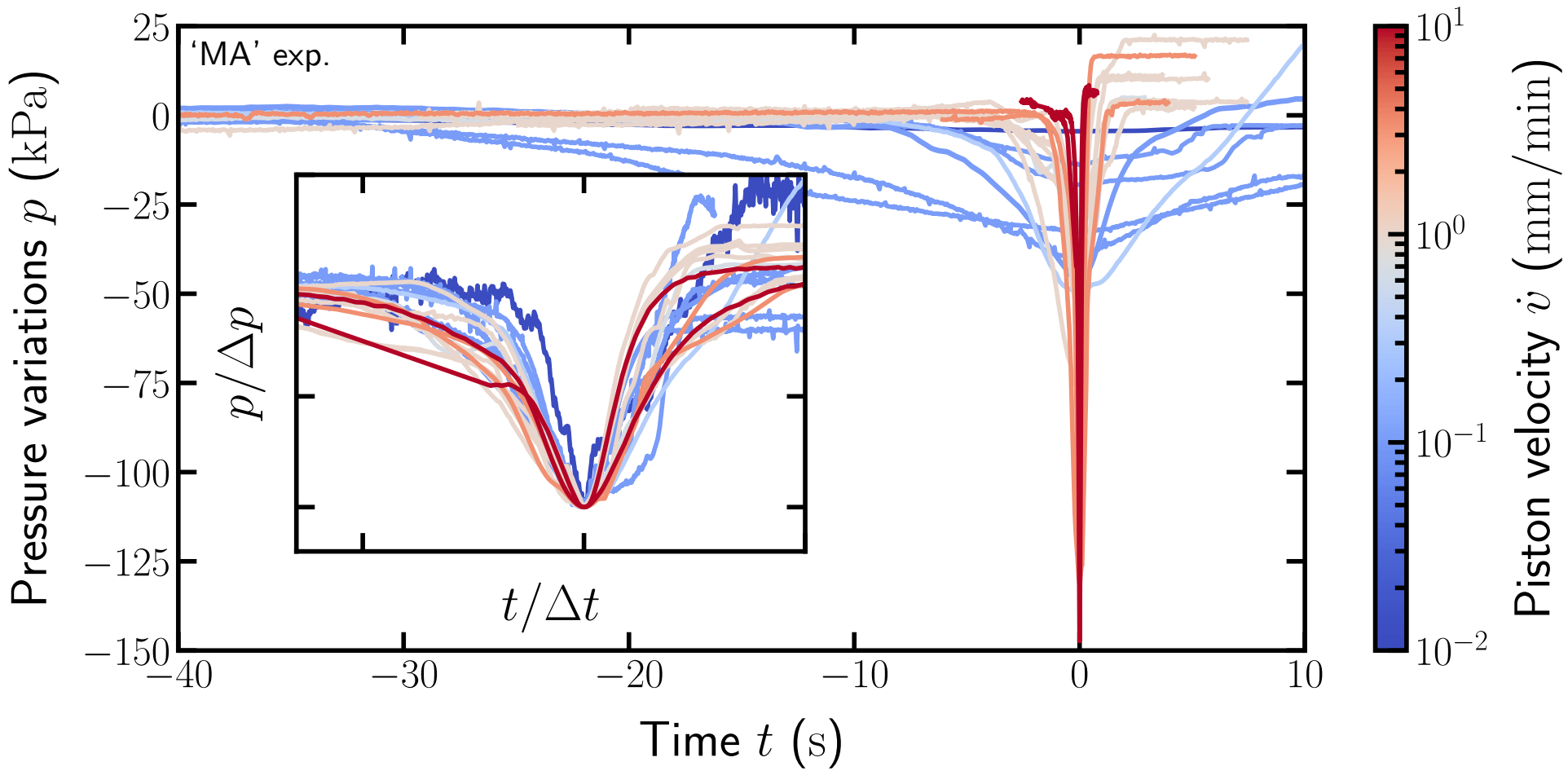

Supplementary Figure 2: Supplementary pore pressure profiles: internal pore pressure measurements of 'MA' experiments.

A.3 Pressure equilibration validation

To ensure measured pore pressure transients were not affected by cell pressure, equilibration times were measured after step changes in confining pressure before the experiments. Internal pressure typically required several minutes to equilibrate (Supplementary Fig. 3), far longer than the < 1 minute fracture tests.

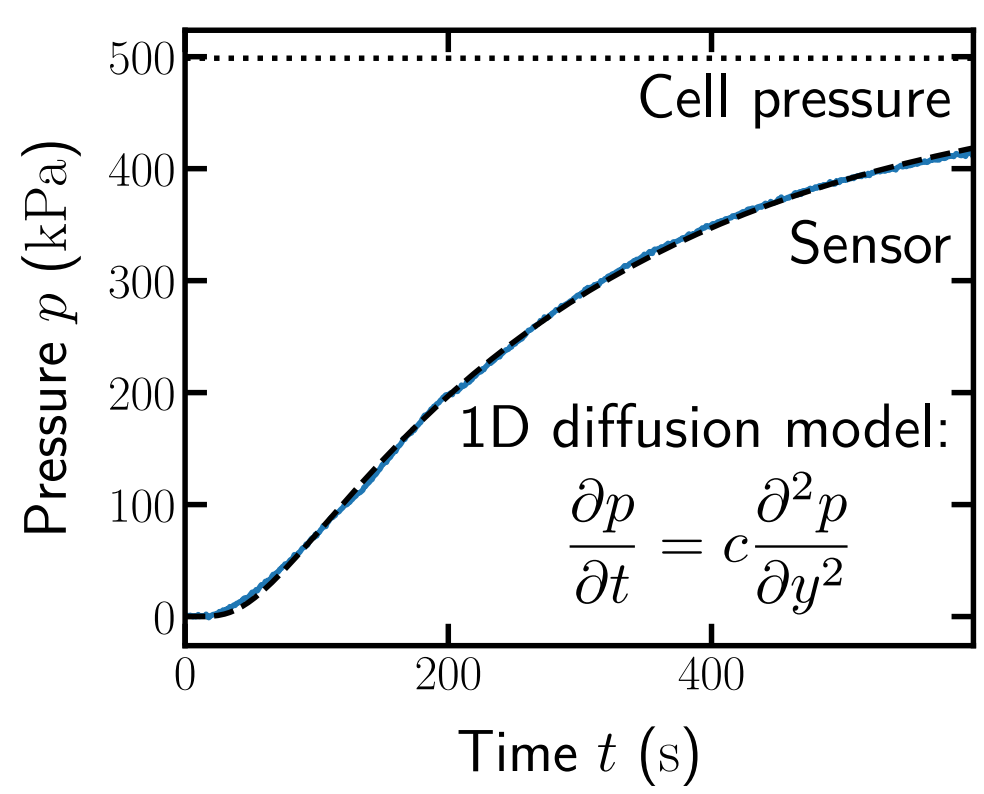

Supplementary Figure 3: Pressure equilibration validation: equilibration of internal pressure at the middle of the specimen after a sudden increase of the pressure of the cell. A 1D diffusion model with a constant pressure on both extremities fits $c = 2.4 \cdot 10^{-7}$ m^2/s.

This response can be modeled as a one-dimensional diffusion process governed by mass conservation and Darcy's law. We fit the cement's diffusivity c using this model to verify the integrity of the internal pressure sensor. For instance, Supplementary Fig. 3 equilibration yields $c = 2.4 \cdot 10^{-7}$ m^2/s within the same order of magnitude as the poroelastic characterization of the cement $c = 7.7 \cdot 10^{-7}$ m^2/s [1].

Notably, many experiments failed due to poor equilibration of pressure caused by: i) seal leakage due to overly viscous glue, ii) blockage of conduit by overly fluid glue, iii) residual air bubbles obstructing pores despite prior vacuum saturation, and iv) grease layers used during casting, which may clog pore openings.

B Mechanical analysis

In all 7 fully instrumented wedge splitting tests (WST) and dry tests, the specimens first exhibited a linear elastic response, with a proportional relation between applied force F and crack mouth opening Δ (Supplementary Fig. 4a and 1b). Once a critical force was reached, crack propagation initiated and the response deviated from linearity. As the crack advanced, F gradually decreased while Δ continued to increase until full propagation. Due to the brittleness of the cement and the positive T-stress in the WST geometry [2], all specimens eventually developed out-of-plane deflections or dynamic events. To maintain validity of the quasi-static, in-plane compliance method, only the portion of data prior to such events was retained.

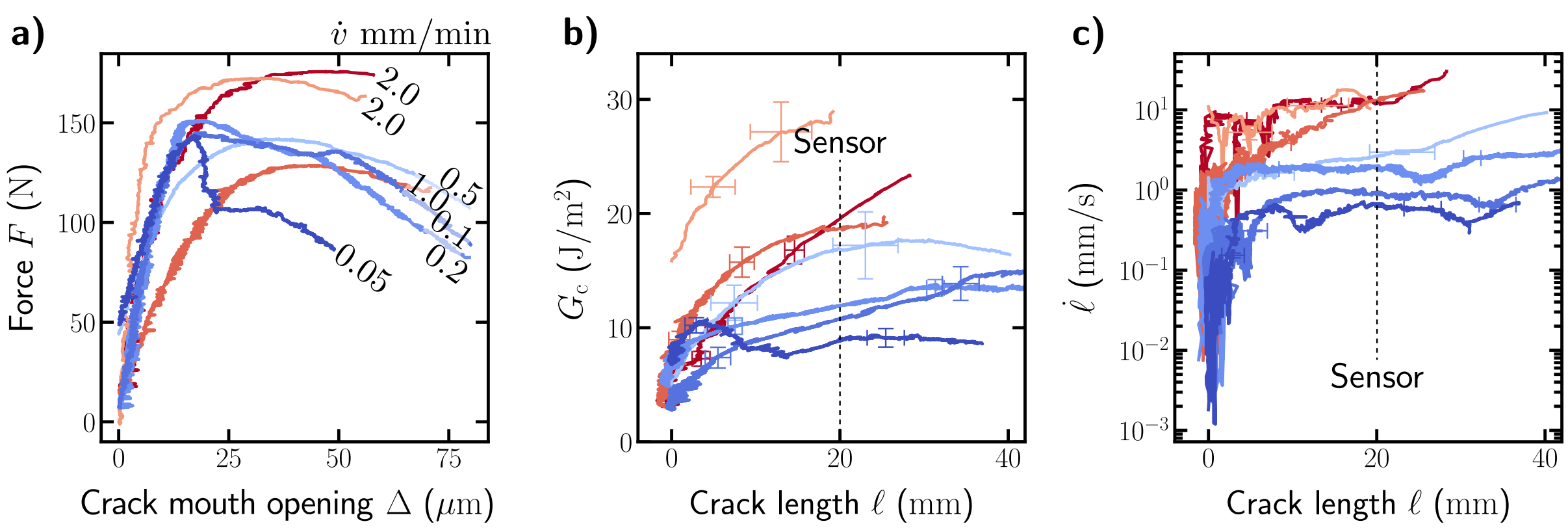

Supplementary Figure 4: Mechanical results from seven fully instrumented WSTs: a) force–opening curves at different piston displacement rates, b) fracture energy G_c, c) crack velocity $\dot{\ell}$ as a function of crack length ℓ. Line colors indicate the plateau crack velocity $\dot{\ell}$. Error bars show propagated measurement uncertainty. Both G_c and $\dot{\ell}$ reach plateaus after ~ 20 mm of propagation, coinciding with the pore pressure sensor position.

From these force–opening curves, fracture energy G_c and crack velocity $\dot{\ell}$ were estimated using a compliance-based method adapted from [3]. The compliance method relates the measured specimen compliance $\Lambda = \Delta/F$ to crack length ℓ through finite element (FE) calibration curves [4]. Half the WST geometry was meshed in Gmsh [5] for varying ℓ (Supplementary Fig. 5a), using second-order T6/Q8 elements with mesh refinement near the crack tip. Linear elastic finite element simulations were run in Cast3M under plane stress, $u = 1$, $E = 1$, and $\nu = 0.3$, representative of the tested cement. The force angle $\theta_F = 15°$ from the wedge was enforced by adjusting the displacement orientation.

The FE analysis produced nondimensional compliance λ and energy release rate g via a J-integral [6] (Supplementary Fig. 5b). Experimental values were obtained by scaling:

$$\Lambda = \lambda E \tag{1}$$

$$G = gEu^2 \tag{2}$$

where E was determined from the elastic portion of the loading curve and u was obtained from the crack mouth opening measurement (with the scaling $u = \Delta/\delta(\ell)$, $\delta(\ell)$ the FE nondimensional opening). Assuming a constant G_c, the FE curves indicate that $\dot{\ell}$ lies between 250 and 1000 times the piston velocity $\dot{v}$ throughout most of the propagation.

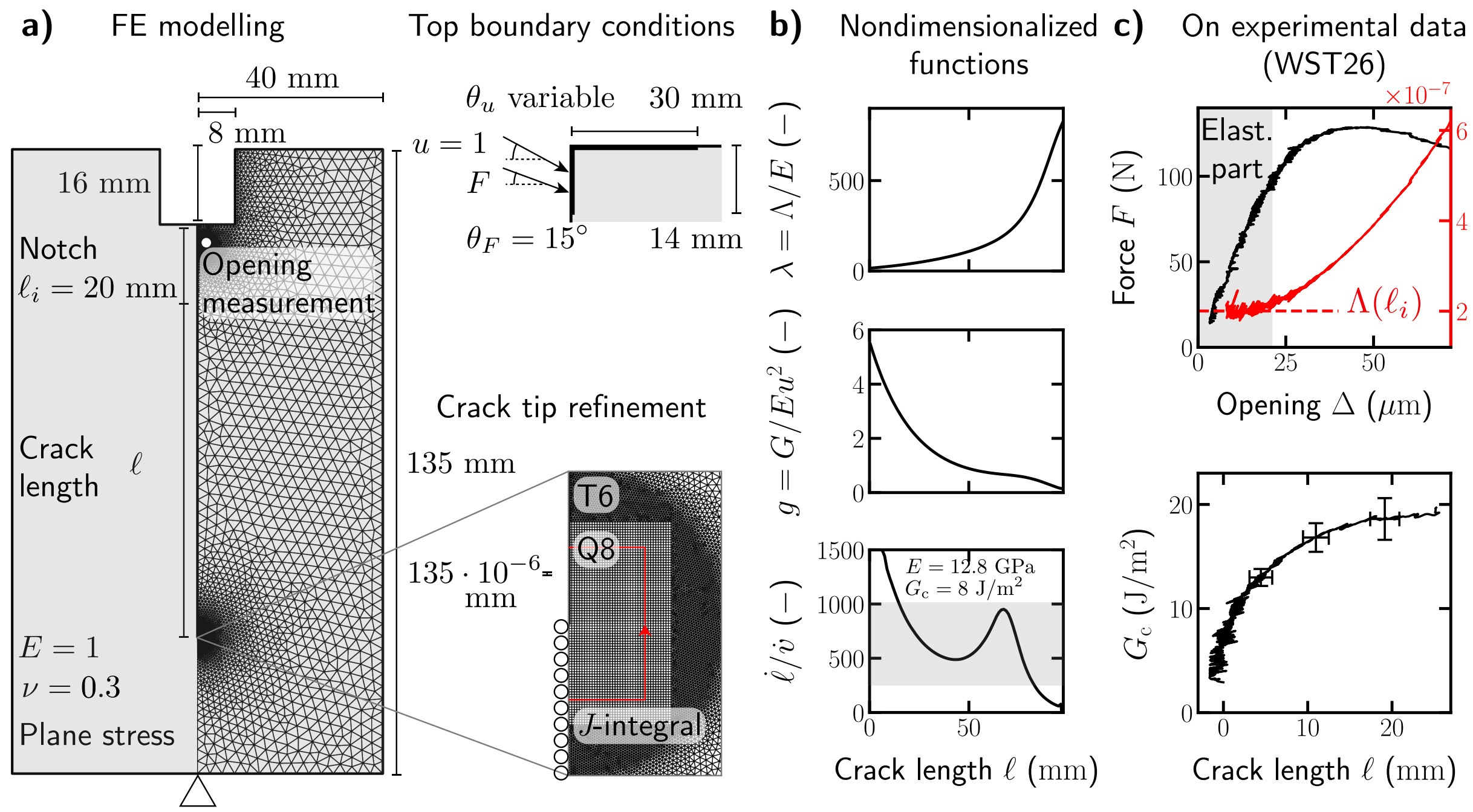

Supplementary Figure 5: Finite element (FE) calibration for the compliance method: a) Half WST geometry meshed with varying ℓ, displacement orientation adjusted to maintain $\theta_F = 15°$. b) Nondimensional compliance λ and energy release rate g from FE simulations, plus $\dot{\ell}$ estimate for constant mechanical properties. c) Example of experimental compliance Λ compared to FE curves to retrieve ℓ and G_c during propagation. Error bars show propagated measurement uncertainty.

From these scalings, we retrieve G and ℓ. Assuming that crack propagation occurs at $G = G_c$, and differentiating in time, we obtain the crack velocity $\dot{\ell}$ (Supplementary Fig. 4b,c). Both quantities increase gradually from the notch and reach plateaus after $\sim$ 20 mm of crack growth — the location of the pore pressure sensor. Plateau crack velocities ranged from $\sim$ 0.5 to 20 mm/s depending on loading rate. Fracture energy increased from initial values near 5 J/m^2 to plateaus between 8 and 26 J/m^2, with a trend of higher G_c at higher $\dot{\ell}$.

Note that, for the dry experiments, the energy release rate G obtained from the compliance method can be directly compared with an intrinsic fracture energy. In the saturated case, this identification is no longer exact. Crack advance modifies the pore-pressure field, and pore pressure enters the fracture problem in two different ways: it changes the stress effectively carried by the solid skeleton near the crack tip, and work is spent to create and relax the pressure gradients generated by pore volume increase. A rigorous poroelastic crack-driving

force would therefore require the measurement of displacement and pressure fields around the crack tip [7,8]. Here we do not attempt to reconstruct this fully-coupled driving force, but rather focus on the equivalent fracture resistance obtained after the pore-fluid response has been lumped into the measured force–crack-length response (see the discussion between J^* and J_e in [9]).

Error propagation using measurement uncertainties ($\sigma_F = 0.1$ N, $\sigma_\Delta = 0.5\,\mu$m, $\sigma_{\ell_i} = 0.3$ mm) yielded maxima of $\sigma_G = 4.0$ J/m^2 and $\sigma_\ell = 4.7$ mm, with average errors $\sim 50\%$ lower. Numerical errors from the FE calibration were negligible in comparison [10].

C Poroelastic framework and scaling analysis

We use a Class G oil well cement with a formulation previously characterized in detail [1,11] (water–cement ratio 0.45, 0.37% dispersant). Here, we summarize the physical parameters governing poroelastic coupling in the present system.

The hydraulic properties are an intrinsic permeability $k \approx 10^{-19}$ m^2 [11], a fluid viscosity $\mu_f = 10^{-3}$ Pa $\cdot$ s, and a fluid bulk modulus $K_f = 2.2$ GPa. The elastic properties are a drained Young's modulus $E = 11.8$ GPa, a drained bulk modulus $K = 8.5$ GPa, and a solid grain bulk modulus $K_s = 21$ GPa [1]. The porosity is $\phi = 0.26$ [1].

The Biot and Skempton coefficients are estimated from standard poroelastic relations [12]:

$$b = 1 - \frac{K}{K_s} \approx 0.6 \tag{3}$$

$$B = \frac{(1/K - 1/K_s)}{(1/K - 1/K_s) + \phi(1/K_f - 1/K_s)} \approx 0.4 \tag{4}$$

The corresponding drained and undrained Poisson's ratios are:

$$\nu = \frac{1 - E/(3K)}{2} \approx 0.27 \tag{5}$$

$$\nu_u = \frac{3\nu + b(1-2\nu)(1-K/K_s)}{3 - b(1-2\nu)(1-K/K_s)} \approx 0.34 \tag{6}$$

C.1 Length scales of physical interactions in cement

Fracture in saturated porous media involves several competing physical mechanisms operating over distinct length scales. We estimate these characteristic scales in the present system to identify the processes governing the experimental response.

Poroelastic diffusion length scale:
The poroelastic length sets the spatial extent over which pore pressure can equilibrate during crack propagation:

$$\ell_{\mathrm{PE}} = \frac{c}{\dot{\ell}} \tag{7}$$

This length scale reflects the competition between hydraulic diffusion, characterized by the poroelastic diffusivity c, and crack advance at velocity $\dot{\ell}$. In isotropic poroelasticity, c is given by:

$$c = \frac{k}{\mu_f}\left[\frac{2E(1-\nu)}{(1-2\nu)}\right]\left[\frac{B^2(1+\nu_u)^2(1-2\nu)}{9(1-\nu_u)(\nu_u-\nu)}\right] \tag{8}$$

For the cement studied here, $c = 7.7 \cdot 10^{-7}$ m^2/s. Combined with $\dot{\ell} = 0.2$–20 mm/s, this yields ℓ_{PE} in the range 0.04–4 mm.

Viscoelastic length scale (creep):
Time-dependent deformation of cement under constant applied stress (creep) can be described by a viscoelastic length scale [13]

$$\ell_{\mathrm{VE}} \propto \frac{\eta\dot{\ell}}{E} \tag{9}$$

with $\eta = 1.56 \cdot 10^{14}$ Pa $\cdot$ s [11] and $E = 11.8$ GPa. This yields $\ell_{\mathrm{VE}} \sim 2.6$–$260$ m, far exceeding all experimental dimensions. The associated relaxation time $\eta/E \sim 10^4$ s is much longer than experimental timescales, indicating that creep is negligible, and the response is effectively elastic.

Inertial effects:
Inertial effects become significant when crack velocities approach elastic wave speeds. In our experiments, crack velocities are 5–6 orders of magnitude lower than the Rayleigh wave speed in cement ($c_R \approx 1.5 \cdot 10^3$ m/s). The fracture process therefore remains quasi-static, and inertial effects can be neglected.

Process zone:
The process zone size, associated with near-tip inelastic deformation and damage processes, is estimated in plane stress as [14]:

$$\xi \propto \frac{1}{2\pi}\frac{EG_{\mathrm{c}}}{\sigma_t^2} \tag{10}$$

Using $E = 11.8$ GPa, $G_{\mathrm{c}} \simeq 9$ J/m^2 (this work), and $\sigma_t \simeq 4$ MPa [15], we obtain $\xi \approx 1.1$ mm, which is of the same order of magnitude as the fracture surface measurements ($\tilde{\xi} \approx 0.6$ mm).

Viscoelastic and inertial effects are negligible, while poroelastic effects remain active at the millimetric scale. In particular, the poroelastic length ℓ_{PE} overlaps the process zone ξ, enabling interactions between fluid flow and near-tip deformation.

C.2 Analytical framework for poroelastic fracture

To interpret these observations, we now compare them to analytical predictions for poroelastic fracture.

Ruina [16, 17] derived a self-similar analytical solution for a tensile crack propagating at constant velocity $\dot{\ell}$ in an infinite, fully saturated poroelastic solid (Biot theory, 2D plane strain, quasi-static crack propagation, impermeable crack faces). In particular, the crack is

loaded by a far-field Mode I stress intensity factor K_{I}. This assumption is appropriate here, as both the process zone ($\xi \simeq 0.6$–0.7 mm) and the poroelastic length ($\ell_{\mathrm{PE}} \simeq 0.04$–$4$ mm) remain small compared to specimen dimensions (80 and 135 mm). In this framework, the competition between crack advance and hydraulic diffusion is captured by the poroelastic length:

$$\ell_{\mathrm{PE}} = \frac{c}{\dot{\ell}} \tag{11}$$

which governs both the amplitude and spatial extent of the pore-pressure perturbation near the crack tip.

Pressure drop:
In the polar coordinate system (r, θ) centered around the moving crack tip, we introduce the normalized radial coordinate $\tilde{r} = r/\ell_{\mathrm{PE}}$. The analytical solution of the pore pressure field p is ([17] Eq. (V.9b), also shown in Main Fig. 1b):

$$p(\tilde{r}, \theta) = \frac{1}{\sqrt{\ell_{\mathrm{PE}}}} \frac{K_{\mathrm{I}}}{\sqrt{2\pi\tilde{r}}} \frac{2B(1+\nu_u)}{3} \left(1 - e^{-\frac{\tilde{r}(1+\cos\theta)}{2}}\right) \cos\left(\frac{\theta}{2}\right) \tag{12}$$

with K_{I} the stress intensity factor. Particularizing Eq. (12) to $\theta = 0$, the amplitude of the pore-pressure drop Δp scales as:

$$\Delta p \sim K_{\mathrm{I}} \sqrt{\frac{\dot{\ell}}{c}}, \tag{13}$$

consistent with our experimental observations. As $K_{\mathrm{I}} \simeq \sqrt{EG_{\mathrm{c}}}$ weakly depends on crack velocity (see Main Fig. 3a), whereas $\dot{\ell}$ spans multiple orders of magnitude, Δp scales as $\sqrt{\dot{\ell}}$.

Attenuation due to dead volume:
We compare the experimental pressure drop with the theoretical pressure drop inferred from Eq. (13) in Supplementary Fig. 6b. The theoretical amplitudes are evaluated using a single representative value of $K_{\mathrm{I}} \simeq 0.44~\mathrm{MPa} \cdot \mathrm{m}^{0.5}$, equal to the mean value estimated from the compliance-based fracture analysis. A linear fit between experimental measurements and theoretical predictions indicates that the measured amplitudes are attenuated by a factor $\mathcal{A} \sim 1/125$ relative to the near-tip predictions. This follows from instantaneous quasi-static partitioning between the poroelastic storage around the crack tip and the relatively large hydraulic compliance of the measurement dead volume (volume of pressure conduit and sensor). Denoting the two compliances C_{PE} and C_{sensor}, the measured drop in attenuation is:

$$\Delta p_{\mathrm{measured}} = \mathcal{A}\Delta p = \frac{C_{\mathrm{PE}}}{C_{\mathrm{PE}} + C_{\mathrm{sensor}}} \Delta p \tag{14}$$

As $\mathcal{A} \approx 1/125$, the drop amplitude is always controlled by the dead volume of the sensor. Because $\mathcal{A}$ is effectively constant over several decades in $\dot{\ell}$, C_{PE} must be approximately velocity-independent. A possible explanation is that C_{PE} is controlled by a finite process zone of enhanced permeability, whose effective storage remains approximately constant.

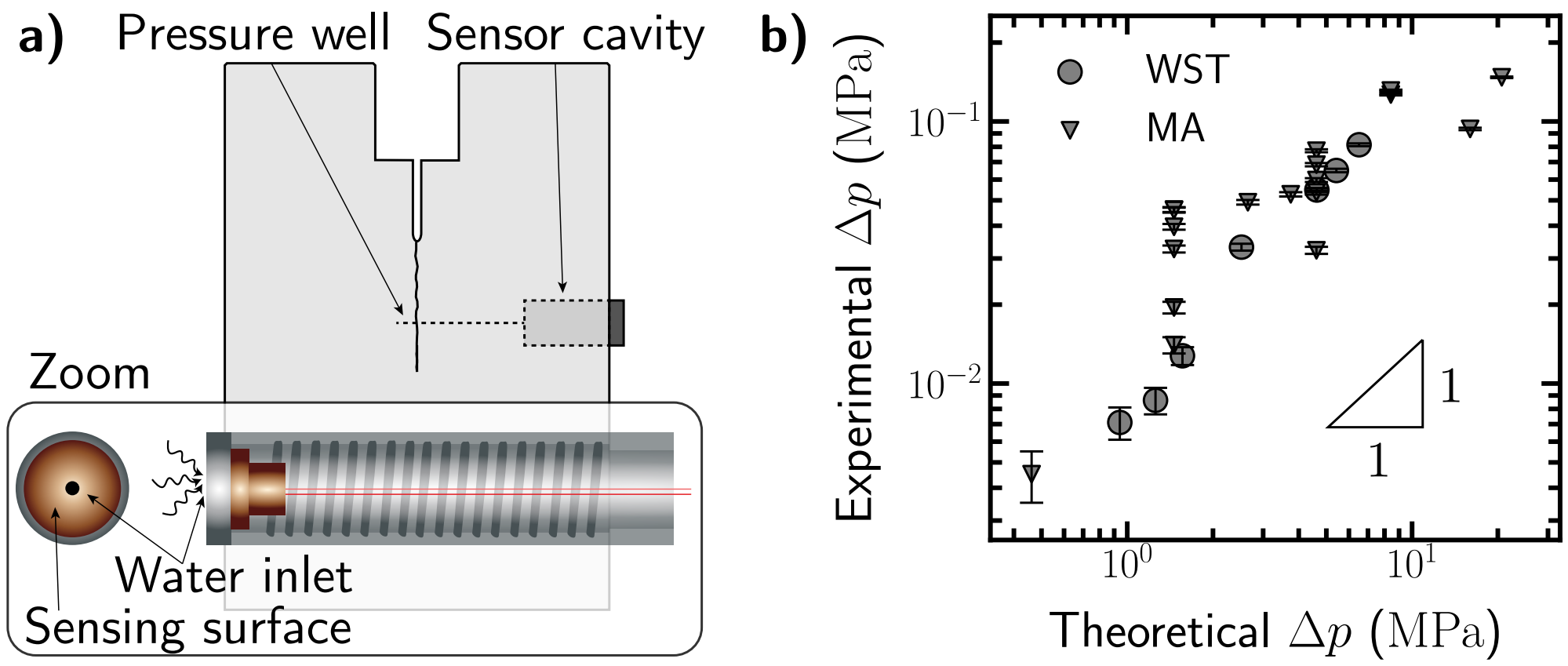

Supplementary Figure 6: Attenuation due to dead volume: a) Schematic of the pressure conduit and sensor design defining the hydraulic dead volume. b) Comparisons between experimental and theoretical predictions (Eq. (12)) of the pressure drop. Experimental values are attenuated by $\mathcal{A} \sim 1/125$. Error bars show measurement uncertainty.

Duration of the pressure drop:
In our experiments, the characteristic duration of the pressure drop simply scales as the transit time of a disturbed zone:

$$\Delta t \sim 1/\dot{\ell} \tag{15}$$

Our measurements are different from the theoretical scaling arising from the transit time of the poroelastic zone $\Delta t \sim \ell_{\mathrm{PE}}/\dot{\ell} \sim c/\dot{\ell}^2$. This discrepancy may be attributed to a combination of the finite size of the specimen, the inelasticity of the material generating a process zone of higher storage capacity, and local permeability changes near the crack tip. Our observed scaling and the fixed value of C_{PE} both indicate that the perturbed area may be of a finite constant size in our experimental setup ($\dot{\ell}\Delta t$ is roughly constant between 10 to 60 mm).

Note that the duration scaling is not influenced by the dead volume of the sensor as its dampening is too fast to affect pressure measurements. Indeed, the sensor and the 20 mm long 0.5 mm conduit act as a hydraulic resistance with a characteristic cutoff time $\tau = R_{\mathrm{sensor}}C_{\mathrm{sensor}}$. Considering a Poiseuille law for the conduit and a conservative estimate of the sensor hydraulic characteristics, it gives $\tau < 10^{-5}$ s which is much below the frequency of acquisition, thus negligible.

Comparison with analytical solution:
To assess how well the analytical solution captures the full temporal evolution of the pressure transient, we compare its normalized form to the experimental signals in Supplementary Fig. 7. However, the experimental scaling for Δt does not follow theoretical predictions, making their comparison difficult. For consistency, the characteristic duration Δt of the analytical solution is determined using the same definition as for the experiments, namely from affine fits to the decreasing and recovery branches of the pressure transient.

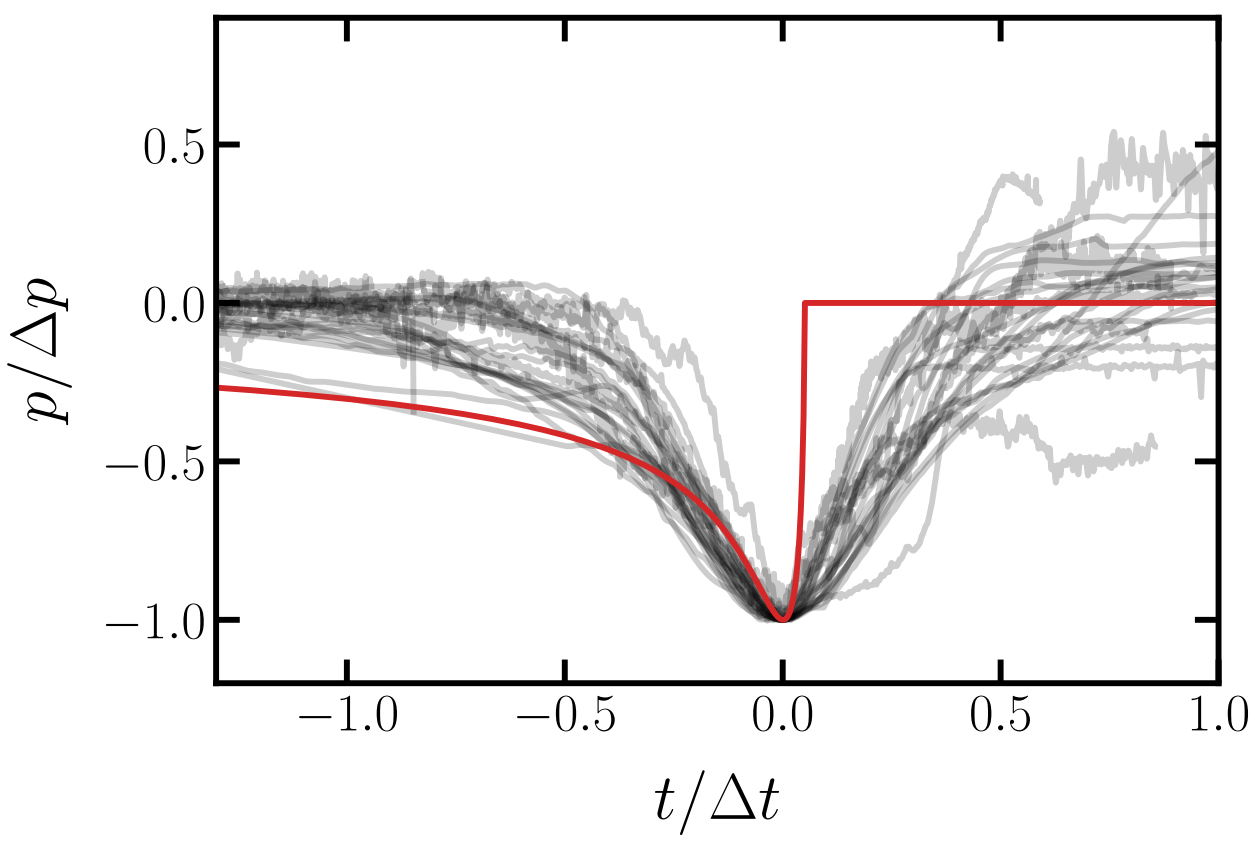

Supplementary Figure 7: Comparison between normalized pressure drops (grey) and Ruina's analytical solution (colored). Theoretical curves are shown with a multiplicative rescaling of the poroelastic length ℓ_{PE}.

While the analytical solution captures the existence of a sharp pressure drop ahead of the crack tip, the theoretical profile exhibits a higher pre-drop level and a sharper recovery than observed experimentally. These discrepancies likely reflect the same finite-size and near-tip effects discussed above, which broaden and smooth the pressure transient.

Cross-relations:
Combining Eq. (13) and (15) allows verifying the integrity of experiments with pore-pressure measurements only. Together, they yield the relationship:

$$\Delta t \sim \Delta p^{-2} \tag{16}$$

All experiments reasonably fit this trend (Supplementary Fig. 8), which validates the experimental design of both fully-instrumented "WST" and pore-pressure only "MA" tests. The minor variations are likely arising due to changes in the system; i.e., cement properties or dead volume variations.

C.3 Toughening regimes

In Ruina's solution, a poromechanical toughening emerges from two mechanisms: (1) a locally drained response at the crack tip, which lowers the stress intensity factor of the crack, and apparently increases the nominal toughness. (2) An effective stress reduction in the process zone. For a fixed yielding stress in the process zone, it increases the nominal toughness. These two mechanisms generate three regimes that are dependent on the interaction of the poroelastic length ℓ_{PE} with the structural draining length ℓ_{struct} (taken here as the specimen half-thickness) and the process zone ξ:

- Regime I (drained), $\ell_{\mathrm{PE}} \gg \ell_{\mathrm{struct}} \gg \xi$:

$$K_{\mathrm{nominal}} = K_{\mathrm{Ic}} \tag{17}$$

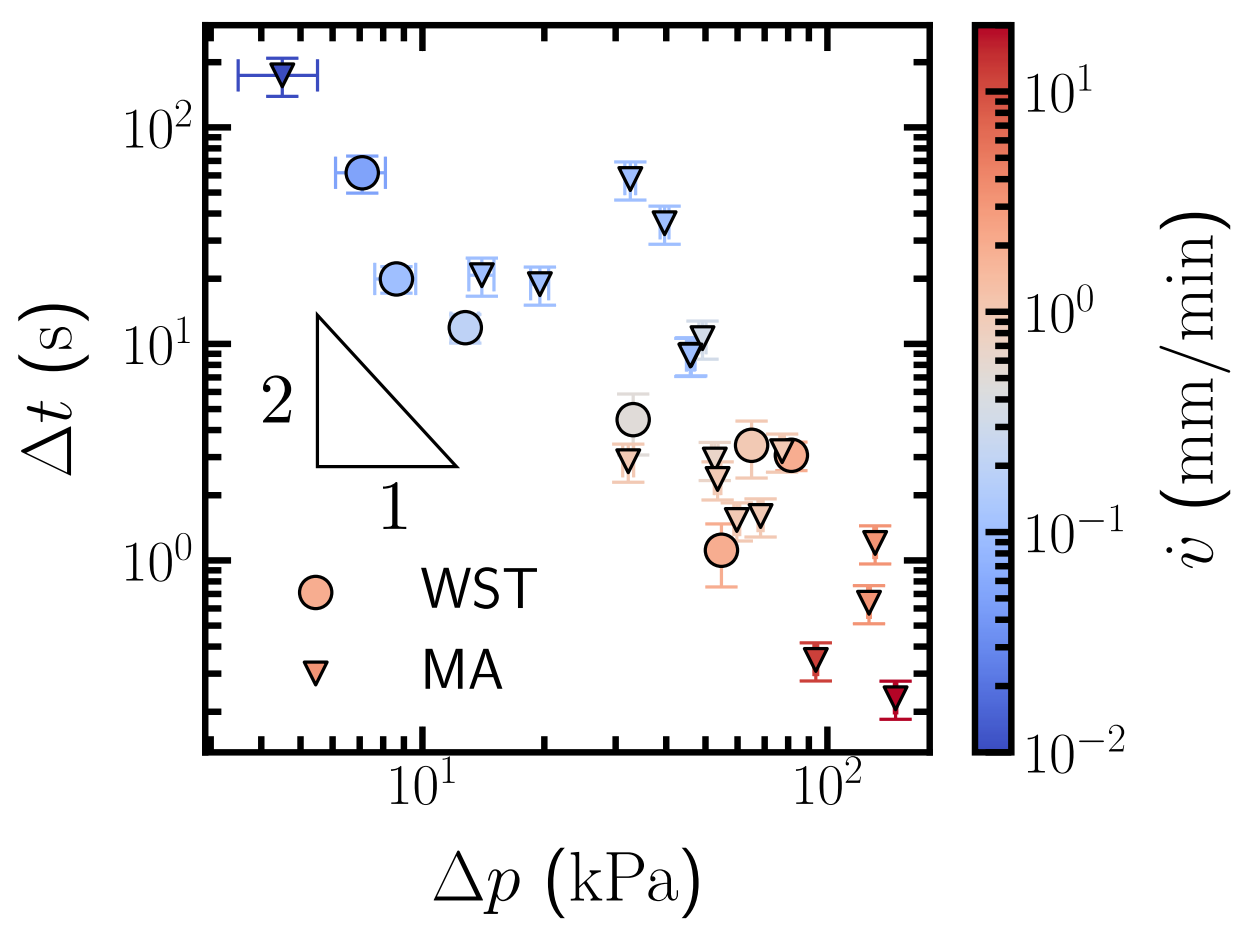

Supplementary Figure 8: Cross-relation between pressure features: Δp and Δt verifying $\Delta t \sim \Delta p^{-2}$ on all experiments. Error bars show measurement uncertainty.

- Regime II (partially undrained), $\ell_{\text{struct}} \gg \ell_{\text{PE}} \gg \xi$:

$$\frac{K_{\text{nominal}}}{K_{\text{Ic}}} = \frac{1-\nu}{1-\nu_u} = \beta \tag{18}$$

Poroelastic constants of petroleum cement were characterized as $\nu = 0.27$ and $\nu_u = 0.34$, which yields a toughening of $\beta \approx 1.11$.

- Regime III (undrained), $\ell_{\text{struct}} \gg \xi \gg \ell_{\text{PE}}$ ([16] Eq. (V.15)):

$$\frac{K_{\text{nominal}}}{K_{\text{Ic}}} = \left(1 - \frac{\nu_u - \nu}{1-\nu}\left[2e^{-\frac{\xi}{\ell_{\text{PE}}}} - \frac{\ell_{\text{PE}}}{\xi}\left(1 - e^{-\frac{\xi}{\ell_{\text{PE}}}}\right)\right] - \frac{2B(1+\nu_u)}{3}\left(1 - e^{-\frac{\xi}{\ell_{\text{PE}}}}\right)\right)^{-1} \tag{19}$$

which describes a sigmoid centered on ξ/ℓ_{PE}. For our petroleum cement with a Skempton coefficient $B = 0.4$, the toughening ranges from $\beta \approx 1.11$ to $3/(3-2B(1+\nu_u)) \approx 1.55$.

As $G_{\text{c}} = K_{\text{Ic}}^2/E'$ [14], these formulae can be directly converted in terms of nominal fracture energy. Regime II gives a toughening of $\beta^2 = 1.23$, and regime III $1.55^2/\beta^2 = 1.96$ for our cement, nearly a two-fold increase.

For water-rich, nearly incompressible materials such as low permeability shales, hydrogels or biological tissues, the combination of $B \to 1$ and $\nu_u \to 0.5$ makes the undrained toughening factor $(3/[3-2B(1+\nu_u)])^2$ very large. As a result, the nominal fracture energy G_c can increase by one to two orders of magnitude relative to the drained value when ℓ_{PE} becomes small (Regime III). These estimates correspond to the ideal undrained limit.

C.4 Applicability and limitations of Ruina's solution

Having established the dominant physical scales, we now discuss the applicability of Ruina's solution to our experimental configuration. Ruina's analytical solution is derived for an infinite, fully saturated poroelastic medium under 2D plane strain conditions, assuming quasi-static propagation at constant crack velocity, impermeable crack faces, and small-scale yielding within a LEFM framework.

In our experiments, these assumptions are only approximately satisfied. The specimens are finite and relatively thin ($h = 20$ mm), the crack velocity fluctuates during propagation, a finite fracture process zone develops near the crack tip, and some fluid exchange may occur along the crack faces.
Nevertheless, several arguments justify the use of Ruina's formulation as a first-order description:

- **Finite size and steady propagation:** Both the poroelastic length ℓ_{PE} and the process zone size remain much smaller than the specimen dimensions, limiting boundary effects. The analysis focuses on the central stage of propagation, when the crack is far from boundaries and the velocity approaches a plateau, approximating Ruina's steady-state assumption. However, the characteristic extent of the measured pressure transients, estimated as $\dot{\ell}\Delta t$, may become comparable to the distance between the pre-crack and the pore-pressure sensor. In addition, the pressure transient begins before steady crack propagation is fully established. These effects may contribute to the discrepancy observed for the duration scaling.

- **Out-of-plane diffusion:** Although the specimen sides are free-draining, measurements are performed near the mid-thickness of the sample. Hydraulic diffusion across half the thickness ($h/2 \approx 10$ mm) has a characteristic timescale $(h/2)^2/c \sim 150$ s, much longer than the fracture durations, limiting transverse drainage effects.

- **Plane stress vs. plane strain:** Our experiments were analyzed assuming plane-stress conditions, while Ruina's solution is derived in plane strain. However, plane stress analyses of steadily-propagating cracks in poroelastic media exhibit the same near-tip field structure, with differences restricted to prefactors [9]. As a result, we expect the scaling of Eq. (13) to hold, while our experimental results exhibit quantitative disagreement with Eq. (12).

- **Hydraulic boundary conditions:** The assumption of impermeable crack faces maximizes the induced pore-pressure perturbation. Allowing fluid exchange along the crack faces reduces the amplitude and alters the distribution of the pressure field [12, 18], so that the actual pressure drop at the crack tip is likely smaller than predicted by Ruina's solution, although the diffusion–fracture coupling remains.

- **Process zone nonlinearity:** Damage within the fracture process zone can locally enhance permeability and alter the near-tip pressure field. Capturing such effects requires cohesive–poroelastic formulations with damage-dependent permeability [19],

beyond the present scaling analysis.

Consequently, Ruina's solution is used here to interpret first-order scaling trends rather than as an exact quantitative prediction.

D Fracture surface analyses

D.1 Surface acquisition

We analyzed post-mortem fracture surfaces from the 7 "WST" fully instrumented wedge-splitting tests and the 3 "Dry" to partially dried reference experiments (Supplementary Fig. 9, 10). They were scanned using a green-light profilometer (Bruker Contour GT-K) over regions of 5×5 mm^2 centered near the pressure conduit. The scanning resolution was 1.97 μm per pixel in both x and z directions. Individual scans were acquired with 50% overlap and subsequently stitched together. Outlier heights, defined as values deviating significantly from the local Gaussian-smoothed surface, were filtered out. Large spherical pores intrinsic to the cement microstructure were also manually filtered. The few missing values ($< 5\%$ of the data) were kept empty to avoid interpolating bias. Prior to roughness analysis, each surface was detrended by subtracting a best-fit quadratic plane to remove any residual tilt or curvature originating from the macroscopic specimen geometry or scanning alignment. The surface of WST07 was scanned in different areas twice as analysis results were uncertain.

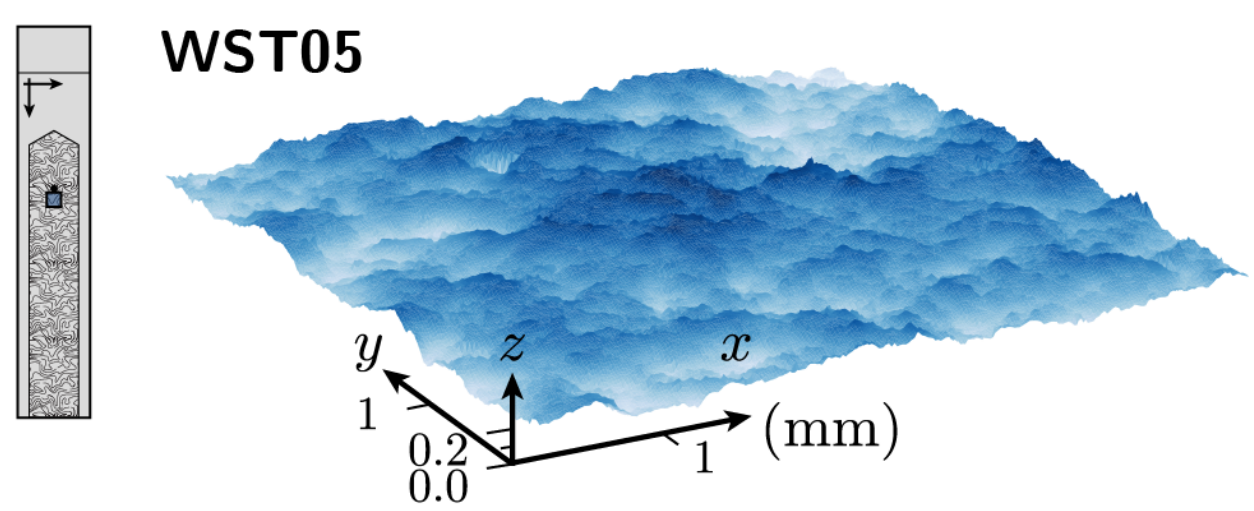

Supplementary Figure 9: Post-mortem fracture surface: location and 5×5 mm^2 profilometric scan of the crack surface near the pore pressure sensor.

D.2 Scale-dependent roughness analysis

The fracture surfaces are indistinguishable when characterized by basic roughness parameters, all exhibiting root-mean-square heights between 40 and 60 μm. However, such global metrics do not capture the scale-dependent nature of the roughness. We therefore analyze the statistics of height increments on the fracture surfaces, defined as:

$$\delta h = |h(\mathbf{x}) - h(\mathbf{x} + \delta \mathbf{x})| \quad \text{with} \quad |\delta \mathbf{x}| = \delta r \tag{20}$$

The probability density functions $P(\delta h \mid \delta r)$ follow a Gaussian distribution for all spatial lags δr (Supplementary Fig. 11). When normalized by their standard deviation, these distributions collapse onto a single curve, indicating self-affine scaling of the form:

$$\delta h \sim \delta r^H \tag{21}$$

where H is the Hurst exponent. However, at small spatial scales, the distributions deviate slightly from a perfect Gaussian collapse, showing flatter tails that indicate more frequent

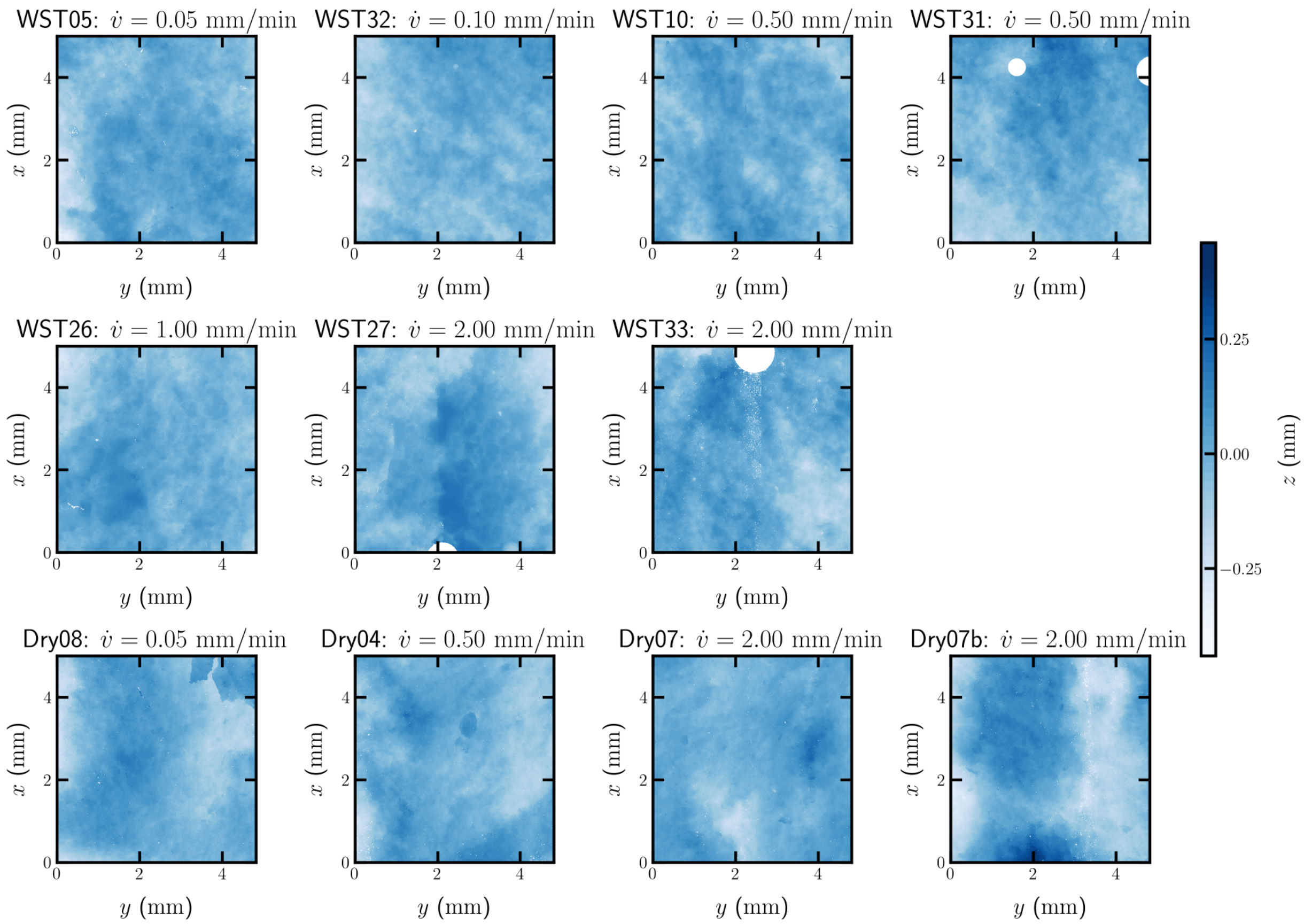

Supplementary Figure 10: Post-mortem fracture surfaces: all scanned surfaces once corrected of their tilt and curvature.

large height fluctuations. This deviation suggests that a single exponent H may not fully capture the complexity of the surface morphology. To characterize this behavior more comprehensively, we evaluate the scaling of the qth-order height fluctuations:

$$\langle |\delta h|^q \rangle^{1/q} = \left\langle \left| h(\mathbf{x}) - h(\mathbf{x} + \delta\mathbf{x}) \right|^q \right\rangle^{1/q}_{|\delta\mathbf{x}|=\delta r} \sim \delta r^{H_q} \tag{22}$$

The dependence of H_q on q reveals whether the surface exhibits a single (mono-affine) scaling behavior with $H_q = H$, or a multi-affine regime where H_q varies with q [20–22].

Our fracture surfaces exhibit both of these behaviors depending on the scale of observation (Supplementary Fig. 14, 15): at fine scales, the surfaces are multi-affine, while they become mono-affine at larger scales.

For instance, WST05 (Supplementary Fig. 14a) illustrates this transition clearly. The height fluctuation functions $\langle |\delta h|^q \rangle^{1/q}$ collapse for all moment orders q when the spatial lag exceeds $\delta r \gg 10^2$ μm. The corresponding local slopes H_q make this collapse more apparent, converging toward a single value at $\tilde{\xi} = 0.6$ mm. At this threshold, $H_q \approx 0.25$, whereas at

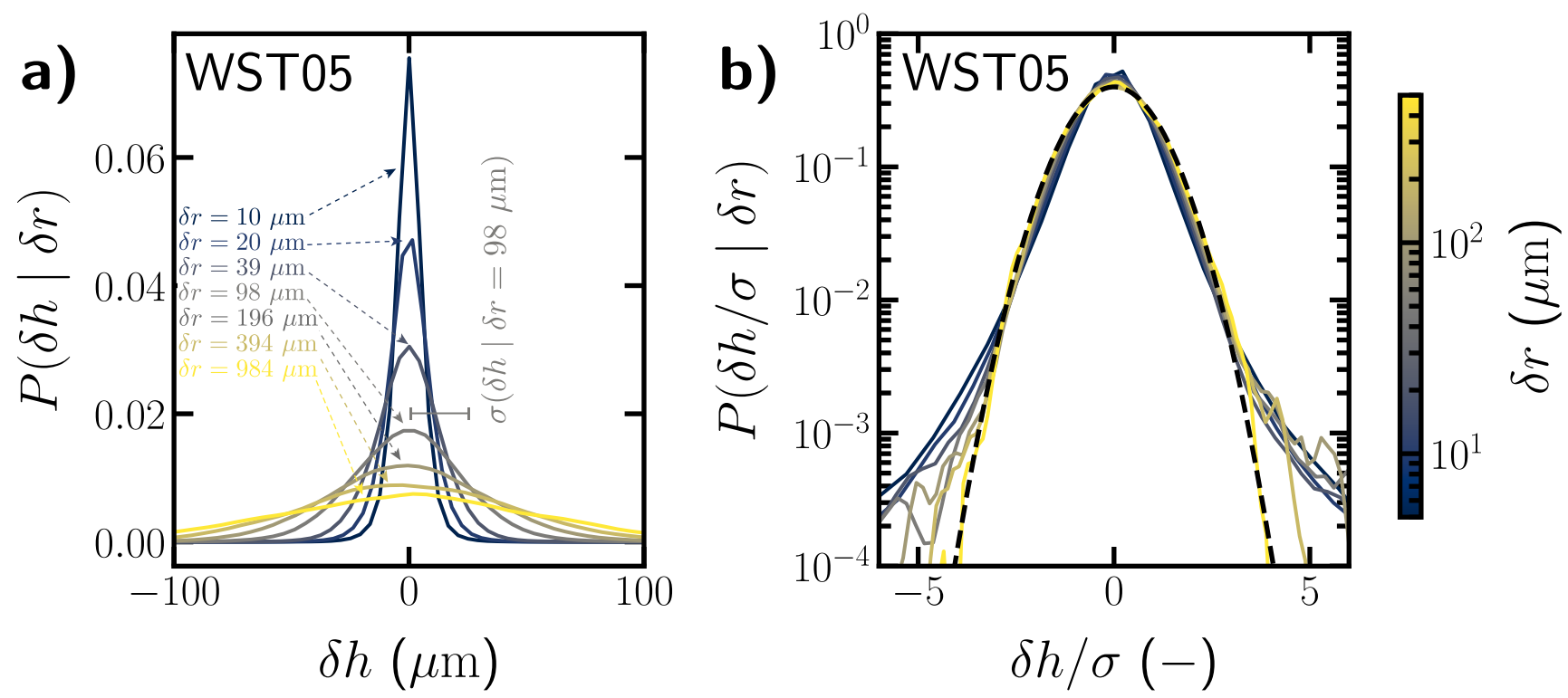

Supplementary Figure 11: Distribution of height fluctuations for a representative fracture surface: a) Height increments $\delta h = h(\mathbf{x}) - h(\mathbf{x} + \delta\mathbf{x})$ computed at different spatial lags $|\delta\mathbf{x}| = \delta r$; b) when normalized by their standard deviation σ, the distributions collapse onto a single Gaussian curve, indicating self-affine scaling of the surface roughness. The log scale highlights deviations at small δr, where flattened tails reveal a transition toward multi-affine behavior at fine scales.

smaller spatial lags, H_q exhibits a broad spectrum that decreases with q, characteristic of a multi-affine regime. All other surfaces exhibit qualitatively similar behaviors with two distinct regimes.

Physically, the highly disordered, multi-affine regime observed at small δr reflects the intermittent and chaotic nature of damage within the fracture process zone, where microcracks nucleate, grow, and coalesce under fluctuating local stresses [21]. At larger scales, these local fluctuations are averaged out, and the crack front advances as an elastically coupled line depinning from the heterogeneous microstructure, producing a mono-affine, self-affine roughness characterized by a single Hurst exponent H [23].

The transition between these two regimes defines a characteristic threshold $\tilde{\xi}$, identified as the crossover scale at which $H_q(\delta r)$ becomes invariant with respect to q. Because $\tilde{\xi}$ marks the boundary between chaotic damage in the process zone and the elastic depinning of the crack front, it provides a physical proxy for the process zone size. As reported in the main article, $\tilde{\xi}$ remains approximately constant between 0.6 and 0.7 mm for dry tests, but decreases systematically from 0.6 to 0.3 mm with increasing crack velocity under saturated conditions.

D.3 Multifractal characteristics

We now examine the multifractal characteristics of the surfaces in the turbulent regime, evaluated at $\delta r = 10\ \mu$m (Supplementary Fig. 12a). Across all experiments, the function H_q decreases systematically with increasing moment order q, from approximately 0.7 at $q = -0.5$ down to 0.2 at $q = 3$ with minimal variations between experiments. The non-unique behavior of H_q reflects the multifractality of the surface roughness: the smallest fluctuations (low q) are smoother, while the rare, large fluctuations (high q) are more intermittent and irregular. The corresponding multifractal spectra $f(\alpha)$, obtained through the Legendre transform $f(\alpha) =$

$q\alpha - qH_q$ with $\alpha = H_q + q\,\partial H_q/\partial q$, collapse remarkably well for all experiments, including the dry references $\delta r = 10$ mm (Supplementary Fig. 12b). These identical statistics within the turbulent regime suggest that the underlying micromechanical processes active within the fracture process zone are universal. They likely reflect diffuse microcracking, damage coalescence, and intermittent deformation events occurring ahead of the crack tip, as observed in quasi-brittle materials such as concrete and mortar [21, 24]. In our case, these processes appear largely unaffected by fluid coupling.

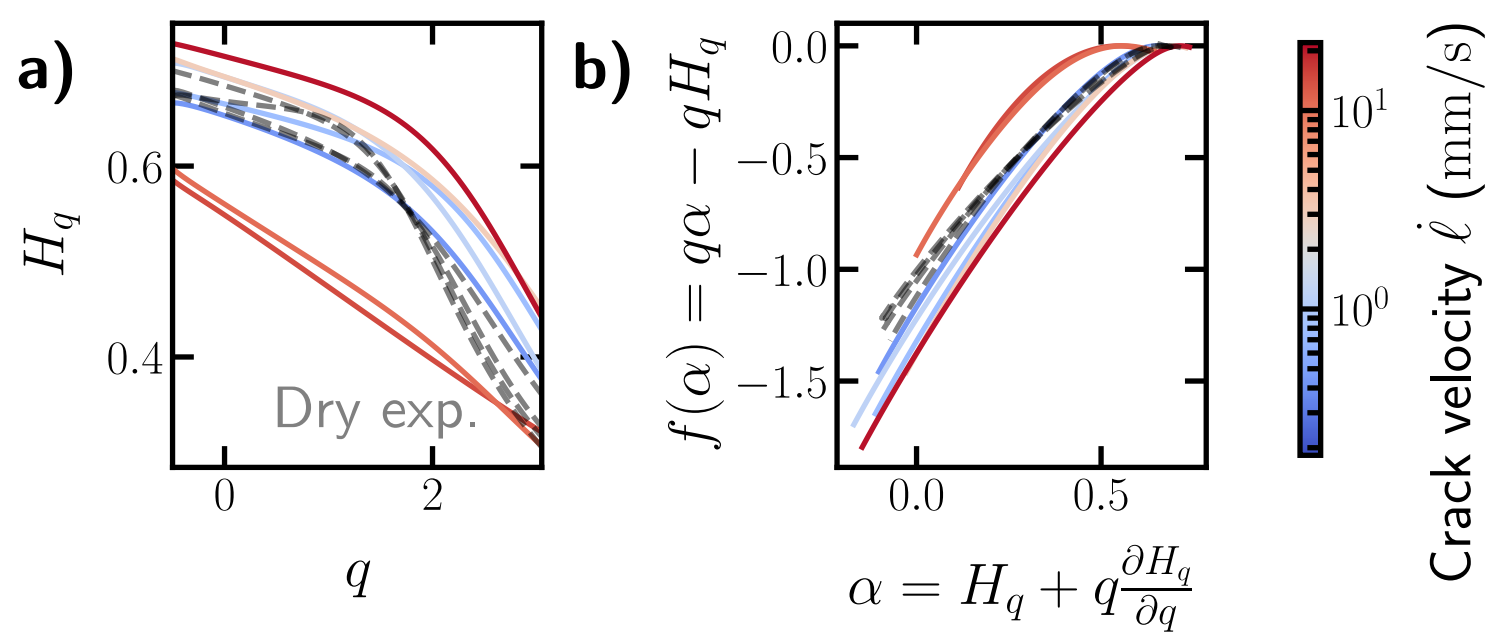

Supplementary Figure 12: Multifractality spectrum (at $\delta r = 10$ mm): a) H_q-q variations in the turbulent regime. b) Legendre transform provides its multifractality spectrum, whose similarities across all experiments suggest nearly identical turbulent mechanisms in the process zone.

Finally, we analyze the evolution of the Hurst exponent in the mono-affine regime, evaluated at $\delta r = \tilde{\xi}$ (Supplementary Fig. 13). In saturated conditions, H increases from approximately 0.25 to 0.5 with crack velocity. In contrast, dry reference experiments exhibit no clear dependence of H on crack velocity, with values scattered around $H \sim 0.4$. These trends correspond to a gradual smoothing of the large-scale roughness that may be caused by a stronger poromechanical coupling. While we propose no model to rationalize the increase in H, the underpressure ahead of the crack tip may alter the effective elastic coupling within the depinning regime.

References

[1] Siavash Ghabezloo, Jean Sulem, Sylvine Guédon, François Martineau, and Jérémie Saint-Marc. Poromechanical behaviour of hardened cement paste under isotropic loading. *Cement and Concrete Research*, 38(12):1424–1437, 2008.

[2] B. Cotterell and J.R. Rice. Slightly curved or kinked cracks. *International Journal of Fracture*, 16(2):155–169, April 1980.

[3] Antoine Guggisberg, Mathias Lebihain, Jo Moore, and Marie Violay. How stress biaxiality controls crack morphology and apparent fracture energy of dikes and sills. *Geophysical Research Letters*, 52(4):e2024GL112638, 2025. e2024GL112638 2024GL112638.

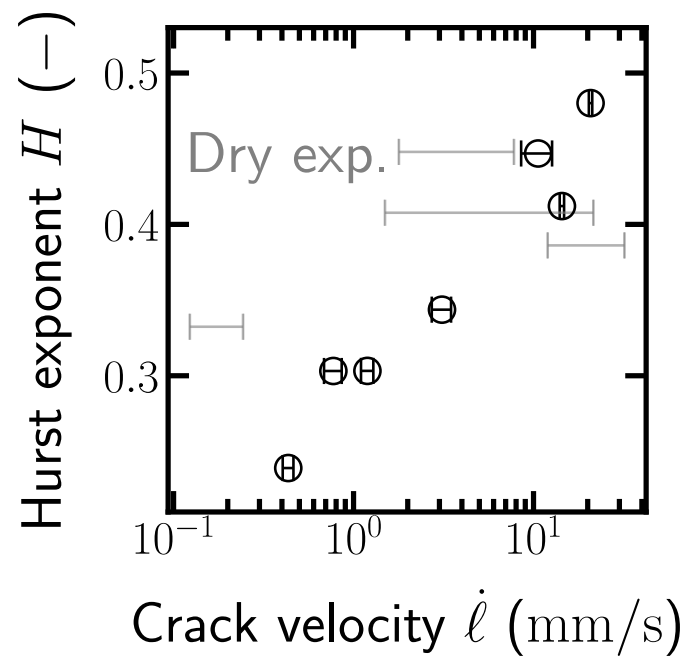

Supplementary Figure 13: Hurst exponent in the mono-affine regime (at $\delta r = \xi$): H increases with crack velocity in saturated experiments, while there is no apparent trend in the scattering of dry experiments. Error bars show propagated measurement uncertainty.

[4] Ashok Saxena and S. J. Hudak. Review and extension of compliance information for common crack growth specimens. *International Journal of Fracture*, 14(5):453–468, October 1978.

[5] Christophe Geuzaine and Jean-François Remacle. Gmsh: A 3-d finite element mesh generator with built-in pre- and post-processing facilities. *International Journal for Numerical Methods in Engineering*, 79(11):1309–1331, 2009.

[6] J. R. Rice. A Path Independent Integral and the Approximate Analysis of Strain Concentration by Notches and Cracks. *Journal of Applied Mechanics*, 35(2):379–386, 06 1968.

[7] Nikolaos Bouklas, Chad M. Landis, and Rui Huang. Effect of solvent diffusion on crack-tip fields and driving force for fracture of hydrogels. *Journal of Applied Mechanics*, 82(8), August 2015.

[8] Luc Dormieux and Eric Lemarchand. Some remarks concerning the propagation of edge cracks in fluid-saturated materials. *Comptes Rendus. Mécanique*, 354(G1):153–163, March 2026.

[9] Yalin Yu, Chad M. Landis, and Rui Huang. Poroelastic effects on steady state crack growth in polymer gels under plane stress. *Mechanics of Materials*, 143:103320, April 2020.

[10] Thiago Melo Grabois, Jan Neggers, Laurent Ponson, François Hild, and Romildo Dias Toledo Filho. On the validation of integrated DIC with tapered double cantilever beam tests. *Engineering Fracture Mechanics*, 191:311–323, March 2018.

[11] Siavash Ghabezloo, Jean Sulem, and Jérémie Saint-Marc. Evaluation of a permeability–porosity relationship in a low-permeability creeping material using a single transient

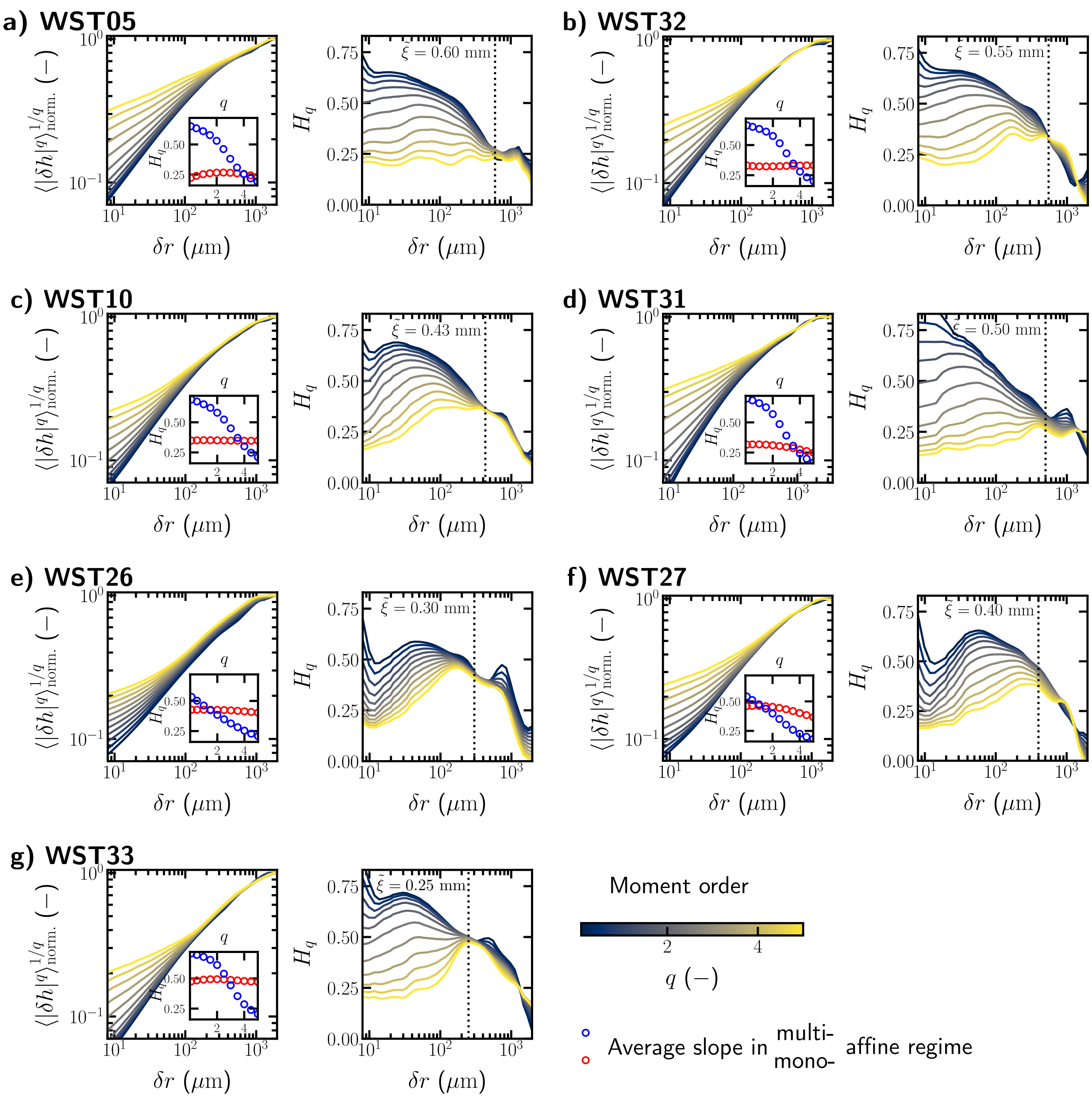

Supplementary Figure 14: Fractal analysis of all saturated tests (a-g).

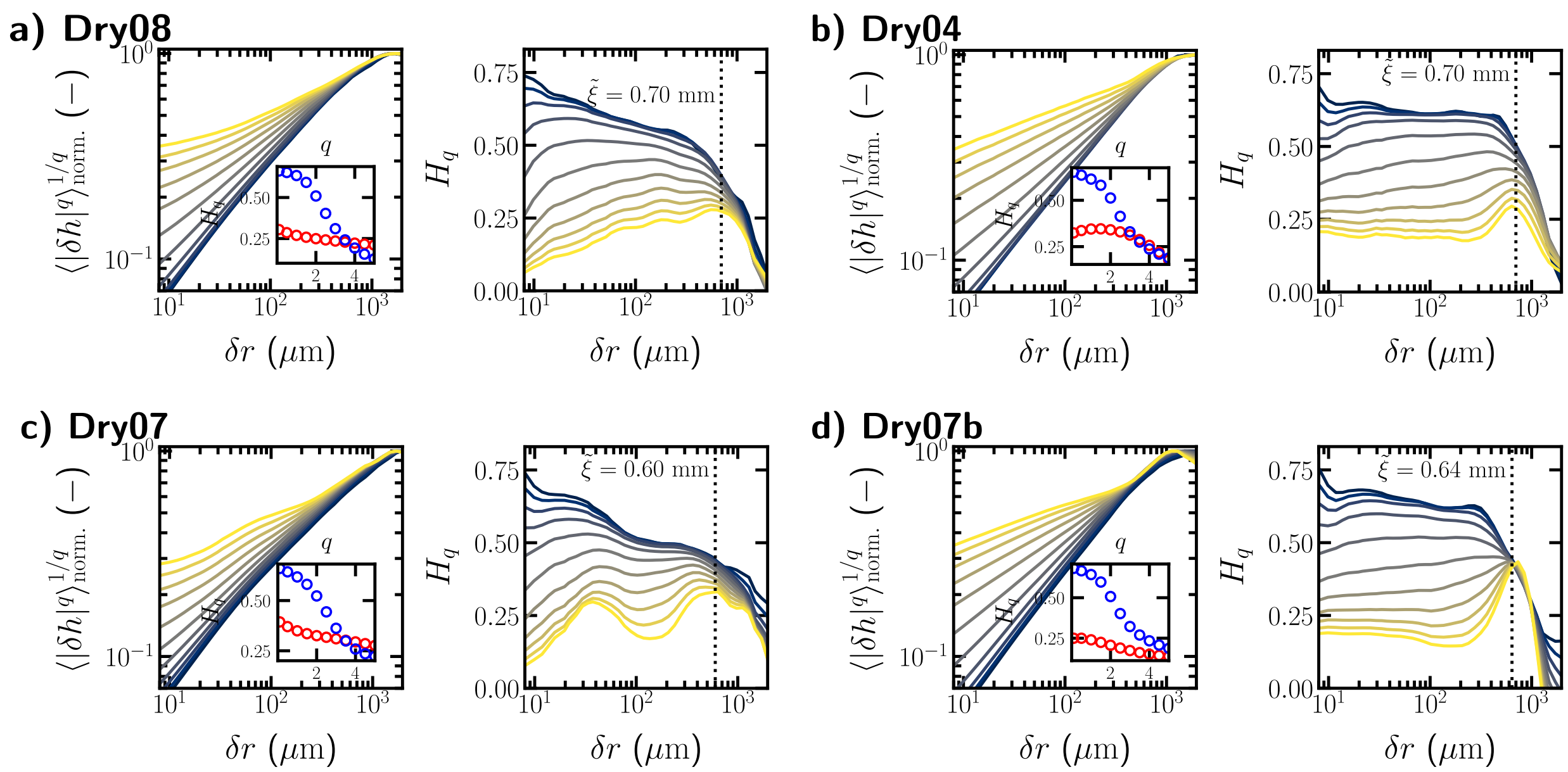

Supplementary Figure 15: Fractal analysis of all dry tests (a-d).

test. *International Journal of Rock Mechanics and Mining Sciences*, 46(4):761–768, 2009.

[12] E. Detournay and Alexander H.-D. Cheng. *Fundamentals of Poroelasticity*, page 113–171. Elsevier, 1993.

[13] Tristan Baumberger and Olivier Ronsin. Environmental control of crack propagation in polymer hydrogels. *Mechanics of Soft Materials*, 2(1), November 2020.

[14] G. R. Irwin. Analysis of Stresses and Strains Near the End of a Crack Traversing a Plate. *Journal of Applied Mechanics*, 24(3):361–364, 06 1957.

[15] Catalin Teodoriu, Zhaoguang Yuan, Jerome Schubert, and Mahmood Amani. Experimental measurements of mechanical parameters of class g cement. In *SPE/EAGE European Unconventional Resources Conference and Exhibition*, 12UNCV. SPE, March 2012.

[16] Andy Ruina. Influence of coupled deformation-diffusion effects on the retardation of hydraulic fracture. In *Proceedings of the 19th U.S. Symposium on Rock Mechanics*, pages 274–282, Stateline, Nevada, May 1978. University of Nevada-Reno.

[17] Andy Ruina. Steady state growth of a mode i fracture in a fluid saturated porous elastic material. Sc.m. thesis, Brown University, Providence, RI, June 1978.

[18] L. Vandamme, E. Detournay, and A. H.-D. Cheng. A two-dimensional poroelastic displacement discontinuity method for hydraulic fracture simulation. *International Journal for Numerical and Analytical Methods in Geomechanics*, 13(2):215–224, March 1989.

[19] Hui Li, Zhenjun Yang, Fengchen An, and Jianying Wu. Simulation of dynamic pulsing fracking in poroelastic media by a hydro-damage-mechanical coupled cohesive phase field model. *Engineering Geology*, 334:107502, May 2024.

[20] Guillaume Mourot, Stéphane Morel, Elisabeth Bouchaud, and Gérard Valentin. Anomalous scaling of mortar fracture surfaces. *Phys. Rev. E*, 71:016136, Jan 2005.

[21] Stéphane Vernède, Laurent Ponson, and Jean-Philippe Bouchaud. Turbulent fracture surfaces: A footprint of damage percolation? *Phys. Rev. Lett.*, 114:215501, May 2015.

[22] Samy Lakhal, Laurent Ponson, Michael Benzaquen, and Jean-Philippe Bouchaud. Wrapping and unwrapping multifractal fields: Application to fatigue and abrupt failure fracture surfaces. *Phys. Rev. Res.*, 7:L012003, Jan 2025.

[23] D. Bonamy and E. Bouchaud. Failure of heterogeneous materials: A dynamic phase transition? *Physics Reports*, 498(1):1–44, January 2011.

[24] A. Carpinteri, G. Lacidogna, and G. Niccolini. Fractal analysis of damage detected in concrete structural elements under loading. *Chaos, Solitons &; Fractals*, 42(4):2047–2056, November 2009.
