# Supplementary material for: Poromechanical toughening in tensile fracture of saturated solids
Source: Nat Commun. 2026 Aug 18;17:9890. doi: 10.1038/s41467-026-76352-3 (PMC13582804; doi:10.1038/s41467-026-76352-3)
Supplement: Supplementary file 2 [file 41467_2026_76352_MOESM2_ESM.pdf]

nature portfolio

Peer Review File

Poromechanical Toughening in Tensile Fracture of Saturated Solids

Corresponding Author: Dr Antoine Guggisberg

This file contains all reviewer reports in order by version, followed by all author rebuttals in order by version.

Version 0:

Reviewer comments:

Reviewer #1

(Remarks to the Author)
This paper aims to bring poromechanical impacts on crack propagation from the realm of the inferred to the observed by measuring pore pressure evolution ahead of a mobile crack tip.

Comments:
1) Line 65ff: It is not clear what about this materials allows to minimize other sources of rate-dependence. Point (ii) is certainly desirable but it is not directly satisfied by high stiffness and/or low permeability. Nor is it clear (iii) why one can assume properties are insensitive to water weakening. It seems this material satisfies objective (i), but (ii) and (iii) are assumed without providing clear evidence to support.
2) Line 103: It might be true that velocities span drained to undrained behavior, but at this point where it is claimed there is not evidence to support. It sounds as if the claim is based on the velocities spanning two orders of magnitude but that fact, in itself, is not sufficient to claim spanning drained to undrained. A brief mention of reference velocity is needed here.
3) Note this range of tip velocities are all probably large enough to preclude creep mechanisms. Class G cement should certainly have large creep strain when loaded slowly enough or for long enough. So, getting to comment 1, the authors should use literature values for Class G (or Class H if Class G is not available) to estimate characteristic creep strain rate and then make the argument that their experiments are in a range where creep strain can be neglected but drain to undrained poromechanical behavior is important.
4) Line 114ff: This is an interesting discussion especially the difference in power law exponent of the scaling. With these being based on scaling laws from diffusion equation, it might be clearer to use the full scaling groups that are dimensionally consistent because changing the power should also imply changes of powers on other governing parameters for this system.
5) Also on the discussion from this paragraph. The attenuation from the dead volume is important. The authors should justify why it is considered to only impact the pressure magnitude and not the temporal evolution (i.e. their Delta T scaling with velocity). It seems the fact that there is a dead volume and a certain rate of fluid flow in and out – there could be an impact on characteristic timescales.
6) The claimed apparent shrinkage of the process zone is very important but the evidence is not presented in a manner that is clear and convincing. Yes, there seems to be a crossover in terms of self-affine statistics, but one has to search the supplementary materials to gain a rough idea and it still seems unclear why that crossover should imply a process zone length. If there is a toughening effect, it seems there could be a simpler explanation in terms of the Irwin length, as the authors discussed for the dry material. This section needs a full revision for clarity and to bring rigor to the discarding of Irwin length and the apparent conjecture that surface roughness in some very fine and subtle detail (it would seem) can lead to such a bold conclusion about how plasticity length scale changes with rate.

Minor:
1) Line 36: Tration->Traction

Reviewer #2

(Remarks to the Author)
The manuscript investigates the fluid pressure ahead of a propagating crack in saturated porous media during wedge splitting tests. The authors confirm a decrease in fluid pressure that is inversely proportional to the square root of the crack

velocity from direct experimental measurements as predicted by the poromechanical analysis.
This pressure drop influences the apparent fracture toughness, which was quantified via crack mouth opening displacement and applied force, and reduces the process zone size, which was inferred through the topological analysis on induced fracture surfaces. Consequently, the findings suggest a velocity-dependent fracture toughness: faster-moving cracks encounter higher resistance than slower ones.
While the experimental validation of this predicted pressure drop is a significant contribution, I have two primary concerns regarding the rigor of the quantitative analysis of the results:
1. Validity of Plane Strain Conditions
For a fracture toughness measurement in the plane strain condition, the specimen thickness (B) must be greater than 2.5 $(K_{IC}/\sigma_T)^2$.
While the manuscript does not report the tensile strength (σ_T) of the material, for cementitious materials, this typically requires a thickness greater than 100 mm. The 25 mm thickness used in this study may be insufficient.
2. Boundary Condition Discrepancies
There is a notable discrepancy between the experimental setup and the theoretical framework cited; Ruina (1978) considers boundary loading applied directly to the crack faces ($0 \le x \le l$, $z=0$) while this study employs a wedge splitting configuration where loading is applied with some angle on ($-P \le x = 0$, $z = \pm P$).
Given the two points raised above, Ruina’s solution may not be quantitatively applicable to this specific experimental configuration. Please provide a formal justification or a comparative analysis (e.g., via numerical modeling or analytical scaling) to demonstrate that Ruina’s solution remains valid despite the distinct boundary conditions and specimen geometry used in your wedge splitting tests.

The following are more detailed comments.
3. Was the crack propagation straight through the thickness (uniform along the y-axis)?
4. Line 101: Fracture energy is computed, not “measured”.
5. Lines 147-148: A fracture does not need to be “cohesive” to have a process zone. A cohesive fracture represents a softening response after the peak load, whereas a process zone can develop before the peak load, still within the realm of LEFM.
6. Line 192, is “B” the Biot coefficient or the Skempton coefficient? In the supplement (Eqs. 5 and 6), it appears to be the Skempton coefficient.
7. Line 192, if B is the Skempton coefficient as described in the supplement, the expression for the effective stress is incorrect.
8. How was the Skempton coefficient measured?
9. Eq. 6, how was K_I obtained (numerically or analytically)?
10. Eq. 8, the value of A is given as 1/125. Is this calculated from C_{PE} and C_{sensor} or inferred from Fig. 11b? If calculated, how were C_{PE} and C_{sensor} obtained?

Reviewer #3

(Remarks to the Author)
Summary
The authors investigate experimentally the effect of poromechanics on mode I fracture propagation. Their Experiments give direct measurements of crack Opening and pore pressure Evolution during fracturing. Their results show the expected localized underpressure ahead of the crack tip and the increase of fracture Energy around the propagating fracture. The authors take great care in comparing their results to analytical solutions and that the boundary conditions do not affect their results.

Overall this is an excellent contribution to the field, with first of its Kind data supporting theoretical findings and thus helping to Advance numerical simulations. The paper is very well structured, well written and higlights clear findings. Nonetheless, I have some comments below, which I would like the authors to treat before publication.

General Comments
The introduction compiles in its first Paragraph a good literature review. However, I think that poroelasticity can actually play an important role not only in hydraulic stimulation but also in natural fractures (See Kamali & Ghassemi and references therein). Regarding this topic and also hydraulic stimulation, the seminal work of Rice & Simons 1976 is, in my opinion also missing in the citations. An slightly smaller role, because it is limited to Mode I fractures and to the lag in the near-tip Region as such might also Play Detournay & Garagash 2003 (although it would fit well with citations 11 - 14). Another work in that direction is the thesis of Kovalyshen, 2010 (which would fit well into the series of simulation papers 19 - 22, and the discussion of Eq. (1) which could be Extended a bit). I would advise the authors to review these contributions and include them approriately, if they think the same way as me. Finally, the authors mention in their Acknowledgements discussions with B. Lecampion. The citet literature 12 and 14 both contain significant typos. B. Lecampion has a paper under review, resolving these typos and additionally explizitely specifing all the relevant kernels for moving singularities in a poroelastic medium. It might be worth to check back with him on this topic.

In the inset of Fig. 2a the match is very very nice. The comparison to [15] in the supplementary material is also ery usefull! It would, in my opinion, be worth to show the scaled version of eq. (6) in the inset Fig. 2a to emphasize the effectively very nice coherence between the experiment and theory. Also, it seems like one experiment (WSP26) has a slightly lower pressure diffusion behind the crack front, do the authors have an explanation for this phenomenon? Interestingly, the same test seems to have a significantly higher apparent fracture energy as expected from theory. As it is not one of the extreme cases, I would

not expect any boundary condition creating this influence.

In my opinion, the explanations of the discrepancy between the scalings of pressure drop duration are not conclusive. As a first mention the authors say, that it is the finite size of the specimen with, thus to my understanding, a strong influence. However, in C.3 (the page after) the authors make a very good and valid claim, as to why the size of the specimen should not induce any boundary effects. I would rather agree, that the inealsticity may play a role. However, these plastic things would most likely be attributed to micro-crack propagation and pore volume expansions, which in turn should not only affect the time scaling but also the pressure drop. So the fact that pressure drop matches so well, while time span does not seems to me difficult to capture in that regard. My guess goes on the third explanation, which is the local permeability changes near the pressure tip. At this point I would refer to my specific comment on the pressure sensor. The drilled pore pressure sensor and it's perturbation to the material around it might be the actual reason why we observe this order of magnitude discrepancy. I can imagine, that this generates a locally higher permeability at this position by inducing preferential flow paths. In fact maybe a strong anisotropy in the hydraulic conductivity at this position, lengthening the pressure drop response? I am curious to know about the author's thinking of this.

How was made sure that the J-integral performed is path Independent? Because this Statement is true for LEFM cracks but not for hydraulic fractures which are not in the toughness dominated regime or where poroelastic components become important. Bouklas & Landis 2015 have developed a methodology for their poro-chemo-mechanical setup. If the base method was used, I would questions the values obtained for their effective path-independence! Because if I understand correctly, teh J-integral is calculated on a modeled dry sample along a given path. The dimensionless values of this path are then used with experimental crack opening (at the same distance to the tip as in the numerical J-integral?) to obtain the experimental G_c. In this configuration, this would be one path evaluated. It is always the same path, so the comparison between experiments is valid, however, it might have a path dependence. I would like the authors to comment on that and maybe try different paths to see if they obtain path independent results.

Specific Comments

P2.L55-59: In this regard of Gels the works of Wei and Kolinski (e.g. Wei et al. 2024) show these mechanisms very well and are a bit more recent than the experiments of Yu and the Landis Group (see e.g. Yu et al. 2018). It needs also to be mentionned, that the Yu papers treat the chemical diffusion, which is an analogy to pore pressure diffusion, but this distinction might be worth mentioning at some point.

P3.L74-76 & P4.L94 & Fig. 5: If I understand correctly, the pore-pressure miniature sensor is placed exactly on the prescribed fracture path. Is the 5mm borehole going down to the crack? Fig. 5 indicates that it is a bigger borehole on the right part of the wedge than towards the crack. This anisotropy in the setup and the borehole as preferential flow-path might influence the pore pressure distribution around the location of the pore pressure sensore. I believe this detail is rather important and would ask the authors to elaborate a bit more on this point.

P4.L111-113: I would be more precise and say that it is the fluid lag of mode I hydraulic fractures. Detournay & Garagash 2003 as well as Kovalyshen, 2010 treat this thematic from an analytical point of view.

P9.L226-231: In my opinion, the ground premise of increased fracture toughness with increased speed is valid. However, as the authors state themselves, the effect requires quite high fracture speeds for geological conditions (P9.L215-216). For larger scale fractures, I would be cautios to say, that such effects rival heterogenity contrasts. In fact, those are two different topics to me. If the toughneing comes from velocity and reaches a limit, the crack will simply slow down and then propagate later slower with the toughness again lower. However, a heterogeneity can not only slow down a fracture but effectively contain it. So the two, in my opinion, should not be treated as rivals. I still think 46 is valid as comparison of order of magnitude, but not really to generate a competition.

P9.L236-248: To me some caution is needed in the conclusions of this section. If the authors talk about tensile cracks due to internal fluid pressure pulses, then such cracks are hardly run-away. At some point, in a confined medium, the internal fluid volume will no longer be sufficient to actually drive fracture propagation. Hydraulic fractures at depth are inherently stable, as they have a component which is volume controlled. However, if combined with mechanical loading, then effectively a tensile runaway crack could emerge. However, these distinctions need to be made very precisely, as else the conclusions are not valid. The statement in the last sentence is correct, if the change runaway crack to large scale tensile fracture. But in the current form I cannot agree.

P9.L254: I would add here the reference to Kanin et al 2020 for backstress and the papers of Yu and Landis for chemical reactions.

Typos, Minor Comments

P7.L166-167: I would slightly reformulate: "... entirely contains the process zone, completely reshaping the failure mechnisms."
P10.L259: Maybe add that this thus requires further Research?

References

Detournay, E., Garagash, D., 2003. The near-tip region of a fluid-driven fracture propagating in a permeable elastic solid. Journal of Fluid Mechanics 494, 1–32

Kamali, A., Ghassemi, A., 2020. On the role of poroelasticity in the propagation mode of natural fractures in reservoir rocks. Rock Mechanics and Rock Engineering 53, 2419–2438.

Rice, J.R., Simons, D.A., 1976. The stabilization of spreading shear faults by coupled deformation-diffusion effects in fluid-infiltrated porous materials. Journal of Geophysical Research 81, 5322–5334.

Yu, Y., Landis, C.M., Huang, R., 2018. Steady-state crack growth in polymer gels: a linear poroelastic analysis. Journal of the Mechanics and Physics of Solids 118, 15–39.

Kovalyshen, Y., 2010. Fluid-Driven Fracture in Poroelastic Medium. Ph.D. thesis. University of Minnesota. Minneapolis, MN.

Wei, X., Li, C., McCarthy, C. et al. Complexity of crack front geometry enhances toughness of brittle solids. Nat. Phys. 20, 1009–1014 (2024). https://doi.org/10.1038/s41567-024-02435-x

Evgenii A. Kanin, Egor V. Dontsov, Dmitry I. Garagash, Andrei A. Osiptsov,
A radial hydraulic fracture with pressure-dependent leak-off,
Journal of the Mechanics and Physics of Solids,
Volume 143,
2020,
104062,
ISSN 0022-5096,
https://doi.org/10.1016/j.jmps.2020.104062.

Bouklas, N., Landis, C. M., and Huang, R. (August 1, 2015). "Effect of Solvent Diffusion on Crack-Tip Fields and Driving Force for Fracture of Hydrogels." ASME. J. Appl. Mech. August 2015; 82(8): 081007. https://doi.org/10.1115/1.4030587

Best Regards
A. Möri

Version 1:

Reviewer comments:

Reviewer #1

(Remarks to the Author)
Thank you for the careful attention to my comments. I am satisfied that the manuscript can be published with the revisions that have been provided.

Reviewer #2

(Remarks to the Author)
Thanks for addressing all my comments thoroughly. I do not have any further remarks except for a minor point.
On comment 2.2, the citation of Ruina (1978) is a proceeding paper at ARMA and does not contain Appendices. Did the authors mean to cite another publication of his e.g., thesis?

Reviewer #3

(Remarks to the Author)
Summary
This is a revised Submission of a manuscript where the authors investigate experimentally the effect of poromechanics on mode I fracture propagation. Their Experiments give direct measurements of crack Opening and pore pressure Evolution during fracturing. Their results show the expected localized underpressure ahead of the crack tip and the increase of fracture Energy around the propagating fracture. The authors take great care in comparing their results to analytical solutions and that the boundary conditions do not affect their results.

Overall this is an excellent contribution to the field, with first of its Kind data supporting theoretical findings and thus helping to Advance numerical simulations. The paper is very well structured, well written and higlights clear findings. I thank the authors for accurately treating the comments of the Reviewers. I believe that the manuscript Quality could be increased with the Revision. I have only three minor comments to be treated.

Typos, Minor Comments
Regarding the Supplementary Material C1. The authors discuss the Poroelastic length scale, which is a such a diffusivity length scale and in many theoretical works mentioned as such (i.e. the works of Cleary and Kovalyshen).
Line 36: tration --> traction (the typo has not been corrected)
Line 739: The Eq. to referenc for the pressure drop is Eq. (14) not Eq. (13).

Open Access This Peer Review File is licensed under a Creative Commons Attribution 4.0 International License, which permits use, sharing, adaptation, distribution and reproduction in any medium or format, as long as you give appropriate credit to the original author(s) and the source, provide a link to the Creative Commons license, and indicate if changes were made.
In cases where reviewers are anonymous, credit should be given to 'Anonymous Referee' and the source.
The images or other third party material in this Peer Review File are included in the article's Creative Commons license, unless indicated otherwise in a credit line to the material. If material is not included in the article's Creative Commons license and your intended use is not permitted by statutory regulation or exceeds the permitted use, you will need to obtain permission directly from the copyright holder.
To view a copy of this license, visit https://creativecommons.org/licenses/by/4.0/

Response to Reviewers

We would like to thank the three reviewers for their thorough and insightful review of the manuscript. Their comments helped us substantially improve the clarity and rigor of the study.

We provide two revised versions of the manuscript: a clean version with final formatting, and a marked version in which all modifications are highlighted in blue. Reviewer comments are reproduced below, followed by our responses in *lavender italic text*.

In response to the reviewers' comments, we substantially revised both the manuscript and the Supplementary Material to clarify the assumptions, limitations, and interpretation of the study. The main changes include:

- the addition of a new Supplementary section quantifying the relevant poroelastic, inertial, and viscoelastic length and time scales,
- clarification of the applicability and limitations of Ruina's asymptotic poroelastic solution for the present experiments,
- clarification of the plane-stress assumptions used in the fracture analysis, the limitations of the classical J-integral in poroelastic fracture, and the interpretation of the measured fracture energy as an *apparent* poromechanical resistance,
- an expanded discussion of the pressure-drop duration scaling discrepancy, including finite-size and transient propagation effects,
- revision of the discussion relating fracture-surface roughness crossover scales to near-tip damage localization and the Irwin length,
- clarification and correction of the poromechanical coefficients and effective-stress notation throughout the manuscript,
- inclusion of several additional references suggested by the reviewers.

Reviewer 1

Major comments

Comment 1.1

Line 65ff: It is not clear what about this material allows minimizing other sources of rate-dependence. Point (ii) is certainly desirable, but it is not directly satisfied by high stiffness and/or low permeability. Nor is it clear (iii) why one can assume properties are insensitive to water weakening. It seems this material satisfies objective (i), but (ii) and (iii) are assumed without providing clear evidence to support.

We revised the manuscript to clarify this point in two ways.

First, we added a dedicated section C.1 "Length scales of physical interactions in cement" in the Supplementary Material (lines 685–716), where we quantify the relevant length and time scales and show that inertial and viscous effects are negligible in our experimental regime, supporting point (ii). In particular, we show that the duration of our fracture experiments is two orders of magnitude shorter than the characteristic time associated with bulk viscous effects, while the rupture velocities explored in this study are 5–6 orders of magnitude lower than the Rayleigh wave speed.

Second, point (iii) is supported directly by our experimental measurements. Comparison between dry and low-velocity saturated tests in Fig. 3a shows no measurable difference in fracture energy. Accordingly, we toned down the corresponding statement in the revised manuscript, which now states that the fracture properties "are shown below to be fairly insensitive to water exposure" (line 70), and explicitly refers to the Results and Supplementary Material C.1 for justification.

Comment 1.2

Line 103: It might be true that velocities span drained to undrained behavior, but at this point where it is claimed there is no evidence to support. It sounds as if the claim is based on the velocities spanning two orders of magnitude but that fact, in itself, is not sufficient to claim spanning drained to undrained. A brief mention of reference velocity is needed here.

We revised the sentence at lines 103–105 to avoid presenting the drained-to-undrained transition as established at this point, and now indicate that the velocity range corresponds to poroelastic lengths relevant to this transition, with the quantitative justification provided later in the Discussion section and Supplementary Material.

> The experiments covered $\dot{\ell} = 0.2$–20 mm/s over two orders of magnitude, corresponding to poroelastic lengths ℓ_{PE} spanning the transition from drained to undrained conditions (Supplementary Material C).

Comment 1.3

Note this range of tip velocities are all probably large enough to preclude creep mechanisms. Class G cement should certainly have large creep strain when loaded slowly enough or for long enough. So, getting to comment 1, the authors should use literature values for Class G (or Class H if Class G is not available) to estimate characteristic creep strain rate and then make the argument that their experiments are in a range where creep strain can be neglected but drain to undrained poromechanical behavior is important.

This point is now addressed in the new section C.1 "Length scales of physical interactions in cement" of the Supplementary Material, where we use literature values [Ghabezloo et al., 2008, 2009] for Class G cement to estimate the relevant viscoelastic scales. We show that the viscoelastic length scale and relaxation time are at least two orders of magnitude larger than the experimental scales, confirming that creep is negligible in our experiments.

We wrote at lines 697–703:

> **Viscoelastic length scale (creep):**
> Time-dependent deformation of cement under constant applied stress (creep) can be described by a viscoelastic length scale [Baumberger and Ronsin, 2020]
>
> $$\ell_{\mathrm{VE}} \propto \frac{\eta \dot{\ell}}{E} \tag{9}$$

with $\eta = 1.56 \cdot 10^{14}$ Pa · s [Ghabezloo et al., 2009] and $E = 11.8$ GPa. This yields $\ell_{\text{VE}} \sim 2.6$–$260$ m, far exceeding all experimental dimensions. The associated relaxation time $\eta/E \sim 10^4$ s is much longer than experimental timescales, indicating that creep is negligible, and the response is effectively elastic.

Comment 1.4

Line 114ff: This is an interesting discussion especially the difference in power law exponent of the scaling. With these being based on scaling laws from diffusion equation, it might be clearer to use the full scaling groups that are dimensionally consistent because changing the power should also imply changes of powers on other governing parameters for this system.

We have revised the manuscript to express the scalings in a dimensionally consistent form, now writing at lines 117 and 123 $\Delta p \sim K_{\text{I}}\sqrt{\dot{\ell}/c}$ and $\Delta t \sim c/\dot{\ell}^2$ following Ruina's solution. This makes the role of other variables explicit.

Comment 1.5

Also on the discussion from this paragraph. The attenuation from the dead volume is important. The authors should justify why it is considered to only impact the pressure magnitude and not the temporal evolution (i.e. their Delta T scaling with velocity). It seems the fact that there is a dead volume and a certain rate of fluid flow in and out – there could be an impact on characteristic timescales.

This point was previously addressed in the subsection "Duration of the pressure drop" of the Supplementary Material C.2 (lines 760–765). We show that the sensor–conduit system has a characteristic response time $\tau < 10^{-5}$ s, which is orders of magnitude smaller than the measured pressure drop durations ($\sim 10^{-1}$–10^{2} s). Therefore, the dead volume affects the pressure amplitude through compliance partitioning, but should not influence the temporal evolution of the signal.

Comment 1.6

The claimed apparent shrinkage of the process zone is very important but the evidence is not presented in a manner that is clear and convincing. Yes, there seems to be a crossover in terms of self-affine statistics, but one has to search the supplementary materials to gain a rough idea and it still seems unclear why that crossover should imply a process zone length. If there is a toughening effect, it seems there could be a simpler explanation in terms of the Irwin length, as the authors discussed for the dry material. This section needs a full revision for clarity and to bring rigor to the discarding of Irwin length and the apparent conjecture that surface roughness in some very fine and subtle detail (it would seem) can lead to such a bold conclusion about how plasticity length scale changes with rate.

We thank the reviewer for this important comment. We revised the structure and arguments of this section for clarity and rigor.

We now explicitly state that $\tilde{\xi}$ is interpreted as a proxy *for the spatial extent of near-tip damage processes, following previous studies on fracture surface roughness and multifractal scaling [Mourot et al., 2005, Vernède et al., 2015, Barak et al., 2019, Aligholi et al., 2022, 2023, Lakhal et al., 2025], rather than as a direct measurement of the process zone size.*

In the revised section, we reorganized the discussion around the comparison with the Irwin length. We first compare dry experiments with the expected Irwin length, showing an order-of-magnitude agreement that supports the use of the fractal crossover scale as a damage-process

proxy. Then, we present the effective-stress argument only as a possible explanation of this apparent localization of damage, rather than as definitive proof of process zone shrinkage. We rewrote the whole subsection at lines 174–203:

> Across all conditions, the height fluctuations on the fracture surfaces exhibit two distinct roughness regimes (Methods; Supplementary Material D), identified from the scale-dependent statistics of height increments. At large (millimetric) scales, the surfaces display a self-affine regime typical of brittle fracture in linearly elastic materials [Ponson et al., 2006]. At smaller scales, however, the roughness becomes increasingly influenced by localized near-tip damage processes, including microcrack nucleation and coalescence [Bouchaud, 1997]. This small-scale regime exhibits complex height fluctuations with fat-tailed statistics [Vernède et al., 2015, Lakhal et al., 2025]. The scale at which these fluctuations become statistically significant defines a characteristic crossover length $\tilde{\xi}$, which we interpret, following previous studies [Mourot et al., 2005, Vernède et al., 2015, Barak et al., 2019, Aligholi et al., 2022, 2023, Lakhal et al., 2025], as a proxy for the spatial extent of damage processes within the process zone.

and

> In dry experiments, $\tilde{\xi}$ remains nearly constant at ~ 0.6–0.7 mm (Fig. 4a), consistent with the expected order of magnitude of the Irwin length in cementitious materials, $EG_{\mathrm{c}}/(2\pi\sigma_{\mathrm{t}}^2) \approx 1.1$ mm [Irwin, 1957], where σ_{t} denotes the tensile strength.

Note that both E and G_{c} are shown to increase with crack velocity, suggesting a contradiction with the observed evolution of $\tilde{\xi}$ using a simple Irwin scaling. However, our effective-stress argument suggests that the apparent tensile strength σ_t is also increased due to pore-pressure shielding ahead of the crack tip.

Minor comments

Comment 1.7

Line 36: Tration → Traction

Addressed.

Reviewer 2

Major comments

Comment 2.1

Validity of Plane Strain Condition:
For a fracture toughness measurement in the plane strain condition, the specimen thickness (B) must be greater than $2.5(K_{\mathrm{Ic}}/\sigma_t)^2$.
While the manuscript does not report the tensile strength (σ_t) of the material, for cementitious materials, this typically requires a thickness greater than 100 mm. The 25 mm thickness used in this study may be insufficient.

We agree that the specimen thickness is insufficient to ensure plane strain conditions, and our experiments are therefore analyzed using plane stress assumptions in the compliance-based analysis. Regarding the applicability of Ruina's solution, we emphasize that we use its near-tip asymptotic formulation, driven by the stress intensity factor K_I. While derived under plane strain conditions, plane stress formulations exhibit the same near-tip structure and scaling laws, differing only by prefactors as shown by Yu et al. [2020]. We therefore use this solution to interpret first-order scaling trends rather than as an exact quantitative prediction. This was further argued in section C.5 of the appendix at lines 832–837.

> **Plane stress vs. plane strain:** Our experiments were analyzed assuming plane-stress conditions, while Ruina's solution is derived in plane strain. However, plane stress analyses of steadily-propagating cracks in poroelastic media exhibit the same near-tip field structure, with differences restricted to prefactors Yu et al. [2020]. As a result, we expect the scaling of Eq. (13) to hold, while our experimental results exhibit quantitative disagreement with Eq. (12).

Comment 2.2

Boundary Condition Discrepancies:
There is a notable discrepancy between the experimental setup and the theoretical framework cited; Ruina (1978) considers boundary loading applied directly to the crack faces ($0 \leq x \leq \ell$, $z = 0$) while this study employs a wedge splitting configuration where loading is applied with some angle on ($-P \leq x \leq 0$, $z = \pm P$).

Ruina [1978] provides both a finite-geometry solution with prescribed boundary loading (Appendix IV) and an infinite-domain near-tip asymptotic solution (Appendix V). We use the latter, in which the crack tip is governed by the universal K_I elastic field and is therefore independent of the specific loading configuration. We clarified and motivated this point in the revised manuscript at lines 722–725.

> In particular, the crack is loaded by a far-field Mode I stress intensity factor K_I. This assumption is appropriate here, as both the process zone ($\xi \simeq 0.6$–0.7 mm) and the poroelastic length ($\ell_{PE} \simeq 0.04$–4 mm) remain small compared to specimen dimensions (80 and 135 mm).

Minor comments

Comment 2.3

Was the crack propagation straight through the thickness (uniform along the y-axis)?

We cannot rigorously demonstrate that the crack front remains perfectly straight in the y-direction. However, the wedge-splitting geometry is close to a double-cantilever-beam configuration, which typically exhibits limited in-plane front deviations [Heinzmann et al., 2024], unlike, e.g., double-torsion tests [Leevers, 1982]. In preliminary tests where the pore-pressure sensor was placed closer to the fracture plane, we observed localized kinking near the sensor, preceded by a nearly straight front segment, supporting the assumption of limited in-plane deviation.

Comment 2.4

Line 101: Fracture energy is computed, not "measured".

We agree with the reviewer. The wording has been revised accordingly at line 102.

Comment 2.5

Lines 147-148: A fracture does not need to be "cohesive" to have a process zone. A cohesive fracture represents a softening response after the peak load, whereas a process zone can develop before the peak load, still within the realm of LEFM.

We agree with the reviewer. We have replaced "cohesive" with "inelastic" at line 153.

Comment 2.6

Line 192, is B the Biot coefficient or the Skempton coefficient? In the supplement (Eqs. 5 and 6), it appears to be the Skempton coefficient.

The notation and expression have been corrected in the revised manuscript. We now distinguish the coefficients explicitly, using common notations in poromechanics: b denotes the Biot coefficient, while B denotes the Skempton coefficient. The expression for the effective stress has been updated accordingly.

Comment 2.7

Line 192, if B is the Skempton coefficient as described in the supplement, the expression for the effective stress is incorrect.

Corrected. The notation and effective stress expression are now clarified consistently throughout the manuscript and Supplementary Material.

Comment 2.8

How was the Skempton coefficient measured?

The Skempton coefficient was not measured directly in this study. Instead, we used material parameters reported for the same cement formulation by Ghabezloo et al. [2008] in their Eq. (50).

In the original manuscript, the values of the Biot and Skempton coefficients were extracted from the thesis manuscript of Ghabezloo [2008]. To improve transparency and consistency with peer-reviewed sources, we now use the properties reported in the later peer-reviewed publications by Ghabezloo et al. [2008, 2009], which were obtained for an identical cement formulation. There, the Biot coefficient is $b \approx 0.6$, and the Skempton coefficient is $B = 0.4$. This leads to a small revision of the Skempton coefficient from $B = 0.44$ to $B = 0.4$, without affecting the conclusions of the manuscript.

We wrote at lines 673–684:

> We use a Class G oil well cement with a formulation previously characterized in detail [Ghabezloo et al., 2008, 2009] (water–cement ratio 0.45, 0.37% dispersant). Here, we summarize the physical parameters governing poroelastic coupling in the present system.
>
> The hydraulic properties are an intrinsic permeability $k \approx 10^{-19}$ m^2 [Ghabezloo et al., 2009], a fluid viscosity $\mu_f = 10^{-3}$ Pa $\cdot$ s, and a fluid bulk modulus $K_f = 2.2$ GPa. The elastic properties are a drained Young's modulus $E = 11.8$ GPa, a drained bulk modulus $K = 8.5$ GPa, and a solid grain bulk modulus $K_s = 21$ GPa [Ghabezloo et al., 2008]. The porosity is $\phi = 0.26$ [Ghabezloo et al., 2008].

The Biot and Skempton coefficients are estimated from standard poroelastic relations [Detournay and Cheng, 1993]:

$$b = 1 - \frac{K}{K_s} \approx 0.6 \tag{4}$$

$$B = \frac{(1/K - 1/K_s)}{(1/K - 1/K_s) + \phi(1/K_f - 1/K_s)} \approx 0.4 \tag{5}$$

Comment 2.9

Eq. 6, how was K_I obtained (numerically or analytically)?

We clarified this point in the revised manuscript. Eq. (6) (now Eq. (13)) is used to derive the scaling relation rather than for direct quantitative prediction. Accordingly, we do not require explicit experimental values of K_I in the scaling analysis:

$$\Delta p \sim K_\mathrm{I}\sqrt{\dot{\ell}/c}$$

Since K_I varies comparatively weakly across the experiments, whereas $\dot{\ell}$ spans multiple orders of magnitude, the scaling is dominated by the $\sqrt{\dot{\ell}}$ dependence, as discussed in the manuscript.

We wrote at lines 732–738:

$$p(\tilde{r}, \theta) = \frac{1}{\sqrt{\ell_\mathrm{PE}}} \frac{K_\mathrm{I}}{\sqrt{2\pi\tilde{r}}} \frac{2B(1+\nu_u)}{3} \left(1 - e^{-\frac{\tilde{r}(1+\cos\theta)}{2}}\right) \cos\left(\frac{\theta}{2}\right) \tag{13}$$

with K_I the stress intensity factor. Particularizing Eq. (13) to $\theta = 0$, the amplitude of the pore-pressure drop Δp scales as:

$$\Delta p \sim K_\mathrm{I}\sqrt{\frac{\dot{\ell}}{c}}, \tag{14}$$

consistent with our experimental observations. As $K_\mathrm{I} \simeq \sqrt{EG_\mathrm{c}}$ weakly depends on crack velocity (see Fig. 3a), whereas $\dot{\ell}$ spans multiple orders of magnitude, Δp scales as $\sqrt{\dot{\ell}}$.

Explicit values of K_I are only used in Fig. 11b to evaluate the theoretical pressure drop. There, we use a single representative value of K_I, taken as the mean value obtained from the compliance-based fracture analysis described in the Methods section. The resulting theoretical pressure amplitudes are then compared with the experimentally measured values.

At lines 737–743:

Attenuation due to dead volume:
We compare the experimental pressure drop with the theoretical pressure drop inferred from Eq. (13) in Fig. 11b. The theoretical amplitudes are evaluated using a single representative value of $K_\mathrm{I} \simeq 0.44$ MPa $\cdot$ m$^{0.5}$, equal to the mean value estimated from the compliance-based fracture analysis. A linear fit between experimental measurements and theoretical predictions indicates that the measured amplitudes are attenuated by a factor $\mathcal{A} \sim 1/125$ relative to the near-tip predictions.

Comment 2.10

Eq. 8, the value of A is given as 1/125. Is this calculated from C_{PE} and C_{sensor} or inferred from Fig. 11b? If calculated, how were C_{PE} and C_{sensor} obtained?

The previous comment also addresses this issue. The attenuation factor $\mathcal{A} \approx 1/125$ is inferred from the fit shown in Fig. 11b, whereas the individual compliances C_{PE} and C_{sensor} were not independently measured. This point has been clarified in the Supplementary Material at lines 737–743.

Reviewer 3

Major comments

Comment 3.1

The introduction compiles in its first paragraph a good literature review. However, I think that poroelasticity can actually play an important role not only in hydraulic stimulation but also in natural fractures (See Kamali & Ghassemi and references therein). Regarding this topic and also hydraulic stimulation, the seminal work of Rice & Simons 1976 is, in my opinion also missing in the citations. A slightly smaller role, because it is limited to Mode I fractures and to the lag in the near-tip region as such might also play Detournay & Garagash 2003 (although it would fit well with citations 11 - 14). Another work in that direction is the thesis of Kovalyshen, 2010 (which would fit well into the series of simulation papers 19 - 22, and the discussion of Eq. (1) which could be extended a bit). I would advise the authors to review these contributions and include them appropriately, if they think the same way as me. Finally, the authors mention in their Acknowledgements discussions with B. Lecampion. The citet literature 12 and 14 both contain significant typos. B. Lecampion has a paper under review, resolving these typos and additionally explicitly specifying all the relevant kernels for moving singularities in a poroelastic medium. It might be worth to check back with him on this topic.

We have added the relevant references throughout the Introduction and Discussion (Kamali and Ghassemi [2020], Kanin et al. [2020a], Detournay and Garagash [2003], Kovalyshen [2010], as well as the recent arXiv manuscript with B. Lecampion [Kanin et al., 2026]). We retained the works of Cleary [1978], Atkinson and Craster [1991] despite known typographical issues, as they remain widely referenced. Because these studies differ substantially in methodology (experimental versus numerical) and physical scope (e.g., reservoir-scale hydraulic fractures versus hydrogels), we incorporated the suggested references at more specific locations in the manuscript rather than consolidating them into a single introductory statement.

We hope these additions improve the context and discussion surrounding Eq. (1).

In the introduction at lines 29-31:

> This interaction occurs in diverse systems, from rocks under hydraulic stimulation [Zoback, 2007, Kanin et al., 2020b, Kamali and Ghassemi, 2020] to gels under tension [Baumberger et al., 2006] and rupturing biological tissues [Dougan et al., 2022], and can exert a first-order control on rupture behavior [Rice and Simons, 1976, Detournay and Cheng, 1991, Bruno and Nakagawa, 1991, Rudnicki, 2001, Kanin et al., 2026].

In the discussion at line 114:

> [...] a zero-pressure gap can develop between the fluid front and the crack tip [Detournay and Garagash, 2003, Kovalyshen, 2010, Bunger et al., 2013].

And at lines 258–259:

> Poromechanical coupling may activate additional mechanisms, e.g. poroelastic back-stress when fluid flow from the fracture to the medium is prevalent [Detournay and Cheng, 1991, Kanin et al., 2020a] [...].

Comment 3.2

In the inset of Fig. 2a the match is very nice. The comparison to [15] in the supplementary material is also very useful! It would, in my opinion, be worth to show the scaled version of eq. (6) in the inset Fig. 2a to emphasize the effectively very nice coherence between the experiment and theory.

We thank the reviewer for this suggestion. We tested the overlay of the scaled form of Eq. (6) (now Eq. (13)) on the normalized pressure transients. While the analytical solution reproduces the existence of a sharp pressure drop ahead of the crack tip, it does not fully capture the shape of the experimental signal. In particular, the theoretical profile exhibits a higher pre-drop level and a sharper recovery than observed experimentally. These discrepancies likely reflect finite-size effects, process-zone effects, and local permeability variations near the crack tip.

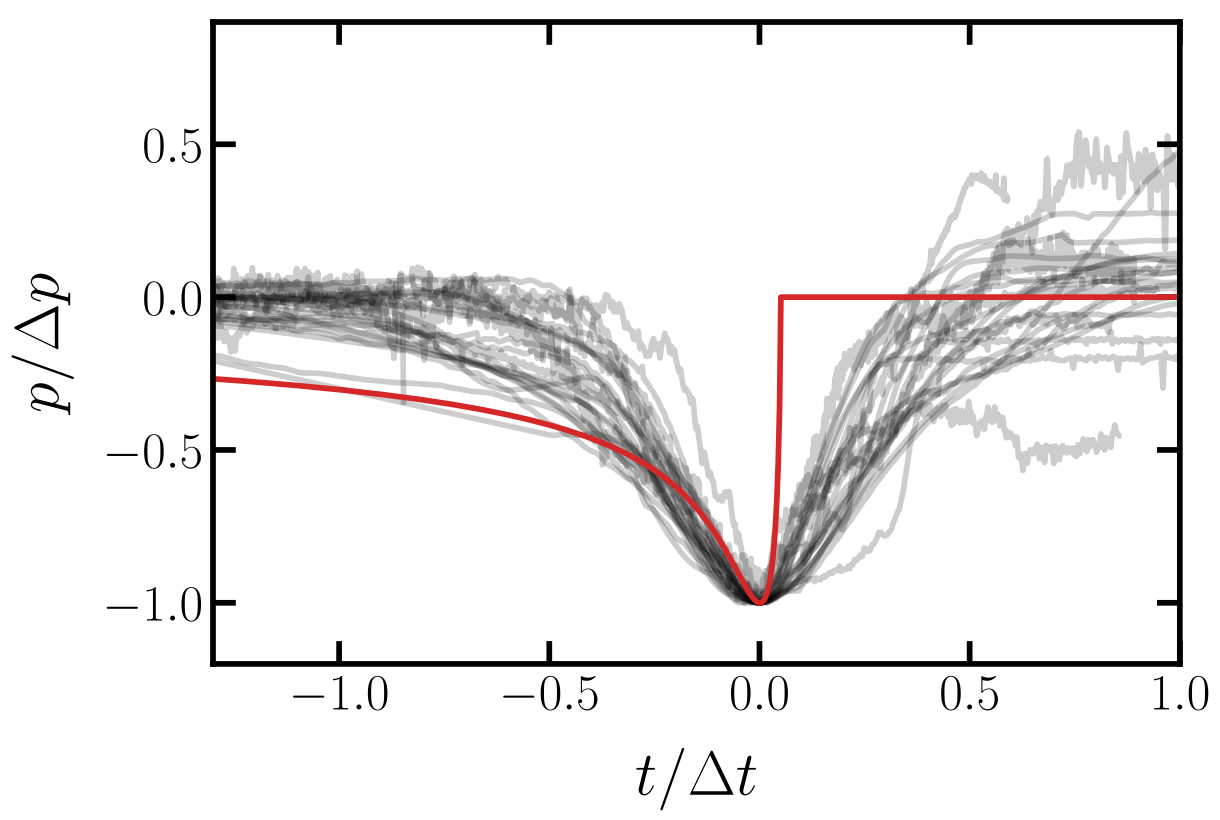

Figure 1: Comparison between normalized pressure drops (grey) and Ruina's analytical solution (red).

We therefore chose not to include this comparison in the main text, to avoid overstating the level of quantitative agreement between theory and experiment. Instead, the comparison is now included in Supplementary Fig. 12 together with a discussion of these possible sources of discrepancy (at lines 766–776).

> **Comparison with analytical solution:**
> To assess how well the analytical solution captures the full temporal evolution of the pressure transient, we compare its normalized form to the experimental signals in Fig. 12. However, the experimental scaling for Δt does not follow theoretical predictions, making their comparison difficult. For consistency, the characteristic duration

Δt of the analytical solution is determined using the same definition as for the experiments, namely from affine fits to the decreasing and recovery branches of the pressure transient.

While the analytical solution captures the existence of a sharp pressure drop ahead of the crack tip, the theoretical profile exhibits a higher pre-drop level and a sharper recovery than observed experimentally. These discrepancies likely reflect the same finite-size and near-tip effects discussed above, which broaden and smooth the pressure transient.

Comment 3.3

Also, it seems like one experiment (WST26) has a slightly lower pressure diffusion behind the crack front, do the authors have an explanation for this phenomenon? Interestingly, the same test seems to have a significantly higher apparent fracture energy as expected from theory. As it is not one of the extreme cases, I would not expect any boundary condition creating this influence.

We thank the reviewer for this observation. The test exhibiting both a lower pressure diffusion behind the crack front and a higher apparent fracture energy corresponds to WST27. This experiment also showed an atypical apparent stiffness compared to the other samples tested under similar conditions.

Because the deviation remains moderate and does not affect the overall scaling trends, we retained this test in the dataset for transparency. Nevertheless, the experiment was repeated at the same loading rate, and the repeated test recovered the general trend observed in the other measurements.

Comment 3.4

In my opinion, the explanations of the discrepancy between the scalings of pressure drop duration are not conclusive. As a first mention the authors say, that it is the finite size of the specimen with, thus to my understanding, a strong influence. However, in C.3 (the page after) the authors make a very good and valid claim, as to why the size of the specimen should not induce any boundary effects. I would rather agree, that the inealsticity may play a role. However, these plastic things would most likely be attributed to micro-crack propagation and pore volume expansions, which in turn should not only affect the time scaling but also the pressure drop. So the fact that pressure drop matches so well, while time span does not seem to me difficult to capture in that regard. My guess goes on the third explanation, which is the local permeability changes near the pressure tip. At this point I would refer to my specific comment on the pressure sensor. The drilled pore pressure sensor and its perturbation to the material around it might be the actual reason why we observe this order of magnitude discrepancy. I can imagine, that this generates a locally higher permeability at this position by inducing preferential flow paths. In fact maybe a strong anisotropy in the hydraulic conductivity at this position, lengthening the pressure drop response? I am curious to know about the author's thinking of this.

We thank the reviewer for this comment. We observe in the new Figure 12, where theoretical predictions of Ruina [1978] are compared to our experimental measurements, that the pore-pressure transient should start much earlier than observed experimentally. At the corresponding time, the crack had not yet reached steady propagation, as assumed in the model of Ruina [1978]. We also compared the distance from the pre-crack to the pore-pressure sensor $L_{\text{sensor}} \simeq 20\,\text{mm}$ *to the typical length scale over which the pore-pressure transient occurs in our experiments* $\dot{\ell}\Delta t$ *(Fig. 2), which ranges from* 10 *to* 60 mm *(average* 28 mm*). Thus, we believe that these finite-size effects are predominant in our experiments.*

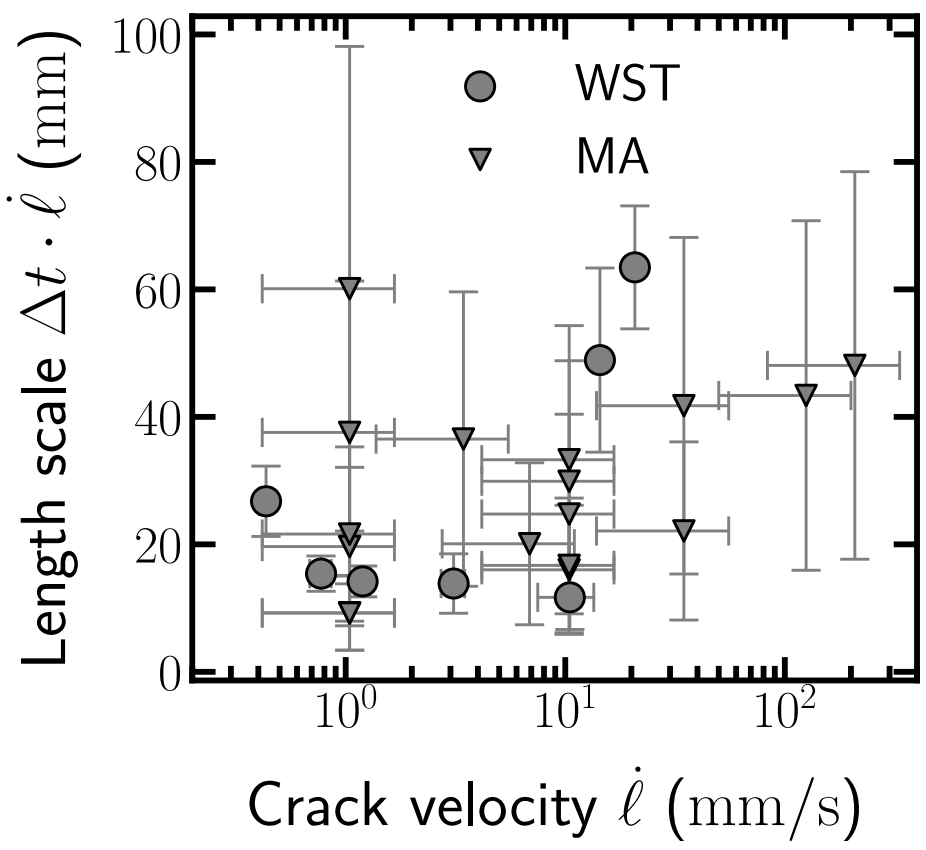

Figure 2: Characteristic spatial extent of the pore-pressure transients, estimated as $\dot{\ell}\Delta t$ for all experiments. $\dot{\ell}\Delta t$ averages 28 mm.

We also investigated the potential influence of the pressure sensor and its associated conduit. In our experiments, the sensor cavity and conduit are cast directly within the specimen using a thin needle and an empty casing, with no post-curing drilling. We therefore do not expect them to induce significant local changes in the cement microstructure or permeability. In addition, a strong preferential flow path from the inside of the triaxial cell to the sensor would lead to near-instantaneous pressure equilibration, which is not the case. Indeed, we carefully verified that pore-pressure equilibration was following a diffusion curve similar to Fig. 8. We therefore consider the sensor influence unlikely to be the dominant origin of the discrepancy.

In section C.4 about the limitations of Ruina's solution, we added in the "Finite size and steady propagation" item at lines 823–827:

> However, the characteristic extent of the measured pressure transients, estimated as $\dot{\ell}\Delta t$, may become comparable to the distance between the pre-crack and the pore-pressure sensor. In addition, the pressure transient begins before steady crack propagation is fully established. These effects may contribute to the discrepancy observed for the duration scaling.

Comment 3.5

How was made sure that the J-integral performed is path Independent? Because this Statement is true for LEFM cracks but not for hydraulic fractures which are not in the toughness dominated regime or where poroelastic components become important. Bouklas & Landis 2015 have developed a methodology for their poro-chemo-mechanical setup. If the base method was used, I would question the values obtained for their effective path-independence! Because if I understand correctly, the J-integral is calculated on a modeled dry sample along a given path. The dimensionless values of this path are then used with experimental crack opening (at the same distance to the tip as in the numerical J-integral?) to obtain the experimental G_c. In this configuration, this would be one path evaluated. It is always the same path, so the comparison between experiments is valid, however, it might have a path dependence. I would like the authors to comment on that and maybe try different paths to see if they obtain path independent results.

We agree that the classical J-integral does not rigorously represent the crack-driving force in a saturated poroelastic medium, as discussed by Bouklas et al. [2015] and Dormieux and Lemarchand [2026]. In the present work, the J-integral is evaluated only within the purely linear elastic

finite-element model used for the compliance method, where the contour integral remains path-independent.

Our objective is therefore not to reconstruct the fully coupled poroelastic driving force, which would require simultaneous measurements of displacement and pore-pressure fields near the crack tip. Instead, we treat the saturated specimen as an equivalent elastic solid and interpret the resulting G_c as an apparent fracture resistance that lumps the additional hydromechanical contributions into the measured force–crack-length response.

We clarified this interpretation in the main text at lines 131–137:

> We now examine how the pressure drop influences the *apparent* fracture energy G_c, obtained from the measured force F and crack length ℓ using the finite-element-based calibration curves (see Supplementary Material B). Here, G_c is not the intrinsic fracture energy of the cement skeleton, but the fracture energy that would be inferred if the saturated specimen were treated as an ordinary elastic solid [Yu et al., 2020]. It therefore lumps the hydromechanical response into a single apparent resistance, accounting for the dissipation generated in creating new surfaces, but also stress shielding by the pore pressure drop and dissipation in the fluid [Bouklas et al., 2015, Dormieux and Lemarchand, 2026].

and in the Supplementary Material B at lines 659–669:

> Note that, for the dry experiments, the energy release rate G obtained from the compliance method can be directly compared with an intrinsic fracture energy. In the saturated case, this identification is no longer exact. Crack advance modifies the pore-pressure field, and pore pressure enters the fracture problem in two different ways: it changes the stress effectively carried by the solid skeleton near the crack tip, and work is spent to create and relax the pressure gradients generated by pore volume increase. A rigorous poroelastic crack-driving force would therefore require the measurement of displacement and pressure fields around the crack tip [Bouklas et al., 2015, Dormieux and Lemarchand, 2026]. Here we do not attempt to reconstruct this fully-coupled driving force, but rather focus on the equivalent fracture resistance obtained after the pore-fluid response has been lumped into the measured force–crack-length response (see the discussion between J^* and J_e in Yu et al. [2020]).

Specific comments

Comment 3.6

L55-59: In this regard of Gels the works of Wei and Kolinski (e.g. Wei et al. 2024) show these mechanisms very well and are a bit more recent than the experiments of Yu and the Landis Group (see e.g. Yu et al. 2018). It needs also to be mentioned, that the Yu papers treat the chemical diffusion, which is an analogy to pore pressure diffusion, but this distinction might be worth mentioning at some point.

We thank the reviewer for pointing this out. We note that Wei et al. [2024] focuses primarily on crack-front geometry effects, whereas Li et al. [2024] addresses poroelastic rupture in hydrogels and was already cited in the original manuscript. To avoid confusion, we clarified at lines 55–56 that the studies of Yu et al. [2019, 2020] involve chemical diffusion, which plays a role analogous to pore-pressure diffusion.

> [...] undrained crack growth manifests as a rate dependence of the fracture energy G_c in experiments [Baumberger et al., 2006, Hassan et al., 2022, Yang et al., 2022, Li et al., 2024], supported by numerical simulations [Noselli et al., 2016, Yang and Lin, 2018, Yu et al., 2019, 2020], where chemical diffusion plays a role analogous to pore-pressure diffusion.

Comment 3.7

L74-76 & P4.L94 & Fig. 5: If I understand correctly, the pore-pressure miniature sensor is placed exactly on the prescribed fracture path. Is the 5 mm borehole going down to the crack? Fig. 5 indicates that it is a bigger borehole on the right part of the wedge than towards the crack. This anisotropy in the setup and the borehole as preferential flow-path might influence the pore pressure distribution around the location of the pore pressure sensor. I believe this detail is rather important and would ask the authors to elaborate a bit more on this point.

The geometry has been clarified in Fig. 5. Only a thin 0.5 mm *conduit intersects the crack path; there is no large borehole connected to the fracture. The sensor and its casing are located approximately* 20 mm *away from the crack.*

Moreover, any significant leakage or preferential flow path would lead to near-instantaneous pressure equilibration. In contrast, all experiments retained in this study exhibit slow equilibration consistent with Fig. 8, ruling out such effects.

The small diameter of the conduit and the distance of the sensor therefore limit its influence on both crack propagation and the local pore-pressure field. The conduit is cast during sample preparation rather than drilled, further minimizing disturbance to the surrounding material.

Comment 3.8

L111-113: I would be more precise and say that it is the fluid lag of mode I hydraulic fractures. Detournay & Garagash 2003 as well as Kovalyshen, 2010 treat this thematic from an analytical point of view.

The text has been revised to specify tensile hydraulic fractures, and the suggested references have been added at line 114.

> [...] a zero-pressure gap can develop between the fluid front and the crack tip [Detournay and Garagash, 2003, Kovalyshen, 2010, Bunger et al., 2013].

Comment 3.9

L226-231: In my opinion, the ground premise of increased fracture toughness with increased speed is valid. However, as the authors state themselves, the effect requires quite high fracture speeds for geological conditions (P9.L215-216). For larger scale fractures, I would be cautious to say, that such effects rival heterogeneity contrasts. In fact, those are two different topics to me. If the toughening comes from velocity and reaches a limit, the crack will simply slow down and then propagate later slower with the toughness again lower. However, a heterogeneity can not only slow down a fracture but effectively contain it. So the two, in my opinion, should not be treated as rivals. I still think 46 is valid as comparison of order of magnitude, but not really to generate a competition.

We agree that velocity-dependent toughening and heterogeneity contrasts should not be treated as equivalent mechanisms. We revised the text accordingly at lines 233–236 to avoid suggesting direct competition, and now only state that toughening variations may influence crack propagation dynamics and stability.

> In geological settings, such toughening variations can be comparable in magnitude to heterogeneity contrasts between lithologies, and may significantly affect crack propagation dynamics and stability [Peruzzo and Lecampion, 2025].

Comment 3.10

L236-248: To me some caution is needed in the conclusions of this section. If the authors talk about tensile cracks due to internal fluid pressure pulses, then such cracks are hardly run-away. At some point, in a confined medium, the internal fluid volume will no longer be sufficient to actually drive fracture propagation. Hydraulic fractures at depth are inherently stable, as they have a component which is volume controlled. However, if combined with mechanical loading, then effectively a tensile runaway crack could emerge. However, these distinctions need to be made very precisely, as else the conclusions are not valid. The statement in the last sentence is correct, if the change runaway crack to large scale tensile fracture. But in the current form I cannot agree.

We agree and have revised the sentence accordingly at lines 250–253, replacing "runaway tensile fracture" with "intermittent ruptures" to reflect volume-controlled conditions.

> As such, short, rapid pressure pulses could use poromechanical toughening to promote distributed microcracking and permeability enhancement while limiting intermittent ruptures, whereas slow or long-duration pressure changes in a more permeable region may instead favor larger crack extensions.

Comment 3.11

L254: I would add here the reference to Kanin et al 2020 for backstress and the papers of Yu and Landis for chemical reactions.

The suggested references have been added at lines 258–261, maintaining a clear distinction between poroelastic backstress and chemically driven effects.

> Poromechanical coupling may activate additional mechanisms, e.g. poroelastic backstress when fluid flow from the fracture to the medium is prevalent [Detournay and Cheng, 1991, Kanin et al., 2020a], fluid-driven advection of reactive species, which can accelerate damage in chemically active systems [Baumberger et al., 2006, Yu et al., 2019, 2020].

Minor comments

Comment 3.12

L166-167: I would slightly reformulate: "... entirely contains the process zone, completely reshaping the failure mechanisms."

The sentence has been reformulated accordingly.

Comment 3.13

L259: Maybe add that this thus requires further research?

A statement highlighting the need for further research has been added at line 264.

> [...] remains largely unexplored in fracture experiments, calling for further investigation.

References

S. Aligholi, L. Ponson, A.R. Torabi, and Q.B. Zhang. A new methodology inspired from the theory of critical distances for determination of inherent tensile strength and fracture toughness of rock materials. *International Journal of Rock Mechanics and Mining Sciences*, 152: 105073, April 2022. ISSN 1365-1609. doi: 10.1016/j.ijrmms.2022.105073.

Saeed Aligholi, Ali Reza Torabi, and Manoj Khandelwal. Quantifying the cohesive strength of rock materials by roughness analysis using a domain based multifractal framework. *International Journal of Rock Mechanics and Mining Sciences*, 170:105492, October 2023. ISSN 1365-1609. doi: 10.1016/j.ijrmms.2023.105492.

Colin Atkinson and R. V. Craster. Plane strain fracture in poroelastic media. *Proceedings of the Royal Society of London. Series A: Mathematical and Physical Sciences*, 434(1892):605–633, 1991. doi: 10.1098/rspa.1991.0116.

Y. Barak, A. Srivastava, and S. Osovski. Correlating fracture toughness and fracture surface roughness via correlation length scale. *International Journal of Fracture*, 219(1):19–30, 2019. ISSN 1573-2673. doi: 10.1007/s10704-019-00377-7.

Tristan Baumberger and Olivier Ronsin. Environmental control of crack propagation in polymer hydrogels. *Mechanics of Soft Materials*, 2(1), November 2020. ISSN 2524-5619. doi: 10.1007/s42558-020-00027-2.

Tristan Baumberger, Christiane Caroli, and David Martina. Solvent control of crack dynamics in a reversible hydrogel. *Nature Materials*, 5(7):552–555, June 2006. ISSN 1476-4660. doi: 10.1038/nmat1666.

Elisabeth Bouchaud. Scaling properties of cracks. *Journal of Physics: Condensed Matter*, 9(21): 4319–4344, May 1997. ISSN 1361-648X. doi: 10.1088/0953-8984/9/21/002.

Nikolaos Bouklas, Chad M. Landis, and Rui Huang. Effect of solvent diffusion on crack-tip fields and driving force for fracture of hydrogels. *Journal of Applied Mechanics*, 82(8), August 2015. ISSN 1528-9036. doi: 10.1115/1.4030587.

M.S. Bruno and F.M. Nakagawa. Pore pressure influence on tensile fracture propagation in sedimentary rock. *International Journal of Rock Mechanics and Mining Sciences &; Geomechanics Abstracts*, 28(4):261–273, July 1991. ISSN 0148-9062. doi: 10.1016/0148-9062(91)90593-b.

Andrew P. Bunger, Elizaveta Gordeliy, and Emmanuel Detournay. Comparison between laboratory experiments and coupled simulations of saucer-shaped hydraulic fractures in homogeneous brittle-elastic solids. *Journal of the Mechanics and Physics of Solids*, 61(7):1636–1654, July 2013. ISSN 0022-5096. doi: 10.1016/j.jmps.2013.01.005.

Michael P. Cleary. Moving singularities in elasto-diffusive solids with applications to fracture propagation. *International Journal of Solids and Structures*, 14(2):81–97, 1978. ISSN 0020-7683. doi: 10.1016/0020-7683(78)90045-8.

E. Detournay and Alexander H.-D. Cheng. *Fundamentals of Poroelasticity*, page 113–171. Elsevier, 1993. ISBN 9780080406152. doi: 10.1016/b978-0-08-040615-2.50011-3.

E. Detournay and D. I. Garagash. The near-tip region of a fluid-driven fracture propagating in a permeable elastic solid. *Journal of Fluid Mechanics*, 494:1–32, October 2003. ISSN 1469-7645. doi: 10.1017/s0022112003005275.

Emmanuel Detournay and Alexander H.-D. Cheng. Plane strain analysis of a stationary hydraulic fracture in a poroelastic medium. *International Journal of Solids and Structures*, 27 (13):1645–1662, 1991. ISSN 0020-7683. doi: 10.1016/0020-7683(91)90067-p.

Luc Dormieux and Eric Lemarchand. Some remarks concerning the propagation of edge cracks in fluid-saturated materials. *Comptes Rendus. Mécanique*, 354(G1):153–163, March 2026. ISSN 1873-7234. doi: 10.5802/crmeca.351.

Carey E. Dougan, Zhaoqiang Song, Hongbo Fu, Alfred J. Crosby, Shengqiang Cai, and Shelly R. Peyton. Cavitation induced fracture of intact brain tissue. *Biophysical Journal*, 121(14): 2721–2729, July 2022. ISSN 0006-3495. doi: 10.1016/j.bpj.2022.06.016.

Siavash Ghabezloo. *Comportement thermo-poro-mécanique d'un ciment pétrolier*. PhD thesis, ENPC, 2008. URL `http://www.theses.fr/2008ENPC0825`. Thèse de doctorat dirigée par Sulem, Jean Géotechnique Paris Est 2008.

Siavash Ghabezloo, Jean Sulem, Sylvine Guédon, François Martineau, and Jérémie Saint-Marc. Poromechanical behaviour of hardened cement paste under isotropic loading. *Cement and Concrete Research*, 38(12):1424–1437, 2008.

Siavash Ghabezloo, Jean Sulem, and Jérémie Saint-Marc. Evaluation of a permeability–porosity relationship in a low-permeability creeping material using a single transient test. *International Journal of Rock Mechanics and Mining Sciences*, 46(4):761–768, 2009. doi: 10.1016/j.ijrmms.2008.10.003.

Sammy Hassan, Junsoo Kim, and Zhigang suo. Polyacrylamide hydrogels. iv. near-perfect elasticity and rate-dependent toughness. *Journal of the Mechanics and Physics of Solids*, 158: 104675, January 2022. ISSN 0022-5096. doi: 10.1016/j.jmps.2021.104675.

Raphael Heinzmann, Rian Seghir, Syed Yasir Alam, and Julien Réthoré. Investigation of fracture source mechanisms through full-field imaging and acoustic emission. *Engineering Fracture Mechanics*, 295:109744, January 2024. ISSN 0013-7944. doi: 10.1016/j.engfracmech.2023.109744.

G. R. Irwin. Analysis of Stresses and Strains Near the End of a Crack Traversing a Plate. *Journal of Applied Mechanics*, 24(3):361–364, 06 1957. ISSN 0021-8936. doi: 10.1115/1.4011547.

Amirhossein Kamali and Ahmad Ghassemi. On the role of poroelasticity in the propagation mode of natural fractures in reservoir rocks. *Rock Mechanics and Rock Engineering*, 53(5): 2419–2438, February 2020. ISSN 1434-453X. doi: 10.1007/s00603-019-02017-x.

Evgenii Kanin, Andreas Möri, Dmitry Garagash, and Brice Lecampion. Steadily moving semi-infinite fracture in plane poroelasticity, 2026. URL `https://arxiv.org/abs/2604.18483`.

Evgenii A. Kanin, Egor V. Dontsov, Dmitry I. Garagash, and Andrei A. Osiptsov. A radial hydraulic fracture with pressure-dependent leak-off. *Journal of the Mechanics and Physics of Solids*, 143:104062, October 2020a. ISSN 0022-5096. doi: 10.1016/j.jmps.2020.104062.

Evgenii A. Kanin, Dmitry I. Garagash, and Andrei A. Osiptsov. The near-tip region of a hydraulic fracture with pressure-dependent leak-off and leak-in. *Journal of Fluid Mechanics*, 892:A31, 2020b. doi: 10.1017/jfm.2020.193.

Yevhen Kovalyshen. *Fluid-driven fracture in poroelastic medium.* Ph.d. dissertation, University of Minnesota, February 2010. URL `https://hdl.handle.net/11299/59581`.

Samy Lakhal, Laurent Ponson, Michael Benzaquen, and Jean-Philippe Bouchaud. Wrapping and unwrapping multifractal fields: Application to fatigue and abrupt failure fracture surfaces. *Phys. Rev. Res.*, 7:L012003, Jan 2025. doi: 10.1103/PhysRevResearch.7.L012003.

P. S. Leevers. Crack-front shape effects in the double torsion test. *Journal of Materials Science*, 17(9):2469–2480, 1982. ISSN 1573-4803. doi: 10.1007/bf00543877.

Chenzhuo Li, Danila Zubko, Damien Delespaul, and John Martin Kolinski. 3d characterization of kinematic fields and poroelastic swelling near the tip of a propagating crack in a hydrogel. *International Journal of Fracture*, 248(1–3):221–235, September 2024. ISSN 1573-2673. doi: 10.1007/s10704-024-00810-6.

Guillaume Mourot, Stéphane Morel, Elisabeth Bouchaud, and Gérard Valentin. Anomalous scaling of mortar fracture surfaces. *Phys. Rev. E*, 71:016136, Jan 2005. doi: 10.1103/PhysRevE.71.016136.

Giovanni Noselli, Alessandro Lucantonio, Robert M. McMeeking, and Antonio DeSimone. Poroelastic toughening in polymer gels: A theoretical and numerical study. *Journal of the Mechanics and Physics of Solids*, 94:33–46, September 2016. ISSN 0022-5096. doi: 10.1016/j.jmps.2016.04.017.

Carlo Peruzzo and Brice Lecampion. Conditions for the local arrest of a hydraulic fracture by a region of larger toughness. *Journal of Applied Mechanics*, 92(10), June 2025. ISSN 1528-9036. doi: 10.1115/1.4068649.

Laurent Ponson, Harold Auradou, Philippe Vié, and Jean-Pierre Hulin. Low self-affine exponents of fractured glass ceramics surfaces. *Physical Review Letters*, 97(12), 2006. ISSN 1079-7114. doi: 10.1103/physrevlett.97.125501.

James R. Rice and Donald A. Simons. The stabilization of spreading shear faults by coupled deformation-diffusion effects in fluid-infiltrated porous materials. *Journal of Geophysical Research*, 81(29):5322–5334, October 1976. ISSN 0148-0227. doi: 10.1029/jb081i029p05322.

JW Rudnicki. Coupled deformation-diffusion effects in the mechanics of faulting and failure of geomaterials. *Applied Mechanics Reviews*, 54(6):483–502, November 2001. ISSN 2379-0407. doi: 10.1115/1.1410935.

Andy Ruina. Influence of coupled deformation-diffusion effects on the retardation of hydraulic fracture. In *19th U.S. Symposium on Rock Mechanics (USRMS)*, pages ARMA–78–0334. American Rock Mechanics Association, May 1978.

Stéphane Vernède, Laurent Ponson, and Jean-Philippe Bouchaud. Turbulent fracture surfaces: A footprint of damage percolation? *Phys. Rev. Lett.*, 114:215501, May 2015. doi: 10.1103/PhysRevLett.114.215501.

Xinyue Wei, Chenzhuo Li, Cían McCarthy, and John M. Kolinski. Complexity of crack front geometry enhances toughness of brittle solids. *Nature Physics*, 20(6):1009–1014, March 2024. ISSN 1745-2481. doi: 10.1038/s41567-024-02435-x.

Chen-Hsueh Yang and Yu-Yun Lin. Time-dependent fracture of mode-i cracks in poroviscoelastic media. *European Journal of Mechanics - A/Solids*, 69:78–87, May 2018. ISSN 0997-7538. doi: 10.1016/j.euromechsol.2017.11.016.

Yan Yang, Haoyu Guo, Zhenjiang Du, Wei Hong, Tongqing Lu, and Tiejun Wang. Rate-dependent fracture of hydrogels due to water migration. *Journal of the Mechanics and Physics of Solids*, 167:105007, October 2022. ISSN 0022-5096. doi: 10.1016/j.jmps.2022.105007.

Yalin Yu, Nikolaos Bouklas, Chad M. Landis, and Rui Huang. Poroelastic effects on the time- and rate-dependent fracture of polymer gels. *Journal of Applied Mechanics*, 87(3), November 2019. ISSN 1528-9036. doi: 10.1115/1.4045004.

Yalin Yu, Chad M. Landis, and Rui Huang. Poroelastic effects on steady state crack growth in polymer gels under plane stress. *Mechanics of Materials*, 143:103320, April 2020. ISSN 0167-6636. doi: 10.1016/j.mechmat.2020.103320.

Mark D. Zoback. *Reservoir Geomechanics*. Cambridge University Press, 2007.

Response to Reviewers

We thank the three reviewers for their final minor comments on the manuscript.

We provide two revised versions of the manuscript: a clean version with final formatting, and a marked version in which all modifications are highlighted in blue. Reviewer comments are reproduced below, followed by our responses in *lavender italic text*. The Supplementary Material is now provided as a separate file.

Reviewer 2

Comment 2.1

On comment 2.2, the citation of Ruina (1978) is a proceeding paper at ARMA and does not contain Appendices. Did the authors mean to cite another publication of his e.g., thesis?

We thank the reviewer for pointing this out. The relevant appendices are contained in Ruina's Sc.M. thesis [Ruina, 1978b], not in the ARMA proceedings paper [Ruina, 1978a]. We have replaced the citation accordingly in the Supplementary Material.

Reviewer 3

Comment 3.1

Regarding the Supplementary Material C1. The authors discuss the Poroelastic length scale, which is a such a diffusivity length scale and in many theoretical works mentioned as such (i.e. the works of Cleary and Kovalyshen).

We agree with the reviewer. We have renamed this quantity the poroelastic diffusion length scale in Supplementary Material C1.

Comment 3.2

Line 36: tration → traction (the typo has not been corrected).

The apparent typo resulted from automatic hyphenation of the word "concentration" in the compiled file. We have adjusted the formatting to avoid this split.

Comment 3.3

Line 739: The Eq. to reference for the pressure drop is Eq. (14) not Eq. (13).

We corrected the equation reference. Following separation of the Supplementary Material into a standalone file, the relevant equation is now numbered Eq. (13).

References

Andy Ruina. Influence of coupled deformation-diffusion effects on the retardation of hydraulic fracture. In *Proceedings of the 19th U.S. Symposium on Rock Mechanics*, pages 274–282, Stateline, Nevada, May 1978a. University of Nevada-Reno.

Andy Ruina. Steady state growth of a mode i fracture in a fluid saturated porous elastic material. Sc.m. thesis, Brown University, Providence, RI, June 1978b.
